# High-harmonic spectroscopy of low-energy electron-scattering dynamics in liquids

**DOI:** 10.1038/s41567-023-02214-0

**Published:** 2023-09-28

**Authors:** Angana Mondal, Ofer Neufeld, Zhong Yin, Zahra Nourbakhsh, Vít Svoboda, Angel Rubio, Nicolas Tancogne-Dejean, Hans Jakob Wörner

**Affiliations:** 1 Laboratory of Physical Chemistry, ETH Zürich, Zürich, Switzerland; 2 Max Planck Institute for the Structure and Dynamics of Matter, Hamburg, Germany; 3 Center for Free-Electron Laser Science, Deutsches Elektronen-Synchrotron DESY, Hamburg, Germany; 4 Department of Physics, Universität Hamburg, Hamburg, Germany; 5 Hamburg Centre for Ultrafast Imaging, Universität Hamburg, Hamburg, Germany; 6 Center for Computational Quantum Physics, The Flatiron Institute, New York, NY USA; 7 Present Address: International Center for Synchrotron Radiation Innovation Smart, Tohoku University, Sendai, Japan

**Keywords:** High-harmonic generation, Attosecond science

## Abstract

High-harmonic spectroscopy is an all-optical nonlinear technique with inherent attosecond temporal resolution. It has been applied to a variety of systems in the gas phase and solid state. Here we extend its use to liquid samples. By studying high-harmonic generation over a broad range of wavelengths and intensities, we show that the cut-off energy is independent of the wavelength beyond a threshold intensity and that it is a characteristic property of the studied liquid. We explain these observations with a semi-classical model based on electron trajectories that are limited by the electron scattering. This is further confirmed by measurements performed with elliptically polarized light and with ab-initio time-dependent density functional theory calculations. Our results propose high-harmonic spectroscopy as an all-optical approach for determining the effective mean free paths of slow electrons in liquids. This regime is extremely difficult to access with other methodologies, but is critical for understanding radiation damage to living tissues. Our work also indicates the possibility of resolving subfemtosecond electron dynamics in liquids offering an all-optical approach to attosecond spectroscopy of chemical processes in their native liquid environment.

## Abstract (web summary)

The application of high-harmonic spectroscopy to liquid samples shows that the cut-off energy is a material characteristic. This approach may also give experimental access to electron mean free paths.

## Main

A prerequisite for accurately interpreting the underlying dynamics of a system using measured high-harmonic spectra lies in formulating a broadly applicable theoretical or conceptual model. In the gas phase, this understanding is often based on the semi-classical three-step model (TSM)^1^ or its quantum mechanical extension^2^, which describes high-harmonic generation (HHG) in terms of a set of electron trajectories initiated by a tunnelling process. The results of this approach are usually in good agreement with those of full ab-initio calculations, and can be used to extract dynamical information from high-harmonic spectroscopy^3,4^. A hallmark of the model is that it correctly predicts the HHG cut-off and its quadratic dependence on both the electric-field amplitude and wavelength, as these depend on the trajectory of the most energetic returning electron trajectory^5,6^. For HHG in crystalline solids, an analogous electron-trajectory picture can, in principle, be applied in momentum space (after applying Bloch’s theorem), which includes both same-site recollision^7^ and coherent scattering from the nearest neighbour atoms^8–15^. The cut-off energy was shown to scale linearly with both field amplitude and wavelength using quantum mechanical calculations, which agree with pioneering experiments^8^ and the momentum-space trajectory picture^7,13,16–20^. However, there still remains some debate about the scaling based on the active HHG mechanisms in different solid systems^21–24^. Moreover, a direct comparison of HHG in crystalline and amorphous solids in the same conditions has shown that there is a steeper efficiency scaling of the extreme ultraviolet harmonics as a function of the driving-field intensity^25^ and a higher cut-off energy^26^ for crystalline materials, pointing to the importance of long-range order in condensed-phase HHG, which has also been explored for one-dimensional models^27,28^.

Electron scattering also affects HHG in exploding plasma droplets^29^. However, the underlying physical mechanism differs from that in the present work (Supplementary Information Section 10). In contrast to gases, crystals and exploding plasma droplets, HHG in the liquid phase is far from being well understood. This is because it is challenging to (1) experimentally measure HHG spectra from bulk liquids, (2) numerically simulate strong-field processes in liquids and (3) formulate an intuitive model that describes non-perturbative light-driven dynamics in liquids. Liquids, therefore, present a unique case in which neither a gas-phase approach (single isolated particle) nor a solid-state approach (Bloch theorem and periodic boundary conditions) is strictly applicable. This gap in knowledge limits potential applications of ultrafast spectroscopy that are especially appealing for liquid targets. Only very recently, by utilizing the flat-jet approach, HHG has been demonstrated in bulk liquids beyond the visible domain^30–32^. However, fundamental questions about the dominant microscopic mechanisms in HHG, the scaling of the cut-off with wavelength and the macroscopic effects still remain unanswered. As most biochemical processes take place in a liquid environment, detailed experimental results and the development of theoretical tools capable of describing the HHG process are crucial for understanding electron dynamics in liquids. Note that our work addresses HHG at typical intensities of ~10^13^ W cm^−^^2^, which are below the optical-breakdown limit and are necessary for opening the true liquid state to high-harmonic spectroscopy. This is in contrast to previous works that studied HHG in exploding plasma droplets far above the optical-breakdown limit^29^ and HHG in the coherent-wake-emission regime at intensities beyond 10^17^ W cm^−^^2^, which has been demonstrated on the surface of liquids^33^. As a consequence of the broken inversion symmetry, both even and odd harmonics were observed in those latter experiments. In contrast, our present experiments probe the bulk of the liquid phase, such that no even harmonics are observed.

Here we experimentally measure high-harmonic spectra from liquid water and alcohols over a broad range of laser wavelengths. We observe that the HHG cut-off energy (*E*_c_), that is, the energy marking the end of the plateau region as defined in the Lewenstein formalism^2^, is wavelength-independent, in strong contrast with the semi-classical TSM for gases^6^, as well as some models for solid-state HHG^7–10,12–14,17^. This implies that potentially new mechanisms are relevant in liquid HHG and that the structural arrangement of the liquid (that is, the lack of long-range order) might play a crucial role in the dynamics. We investigate this experimental result with a combination of newly developed ab-initio techniques and introduce a semi-classical model for HHG in liquids. Our proposed model takes electron scattering into account and successfully reproduces the observed wavelength-independence of *E*_c_. We identify a key parameter in HHG from the liquid phase—the effective mean free path (*λ*_MFP_)—which we extract from measurements using the extended semi-classical model.

**Fig. 1:**
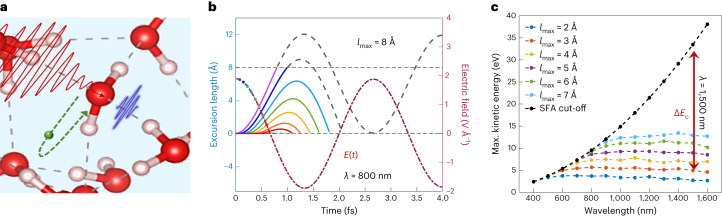
Effect of electron scattering on HHG spectra in liquids. **a**, Schematic illustration of the extended trajectory-based semi-classical model. An electron (green) is ionized by the laser field, accelerated and then either recombines directly with its parent ion (dashed green arrow) or scatters off another molecule. **b**, Trajectories of returning electrons from the standard TSM within an 800 nm driving laser field of 1.9 V Å^−1^. The dots on the electric field (red dashed-dotted line) represent the ionization times of the electrons (the colour of a dot corresponds to the colour of the respective trajectories). The horizontal grey dashed line denotes the limited excursion length (*l*_max_) of 8 Å, imposed by scattering. **c**, Wavelength-scaling of *E*_c_ in the absence (standard TSM: black dashed line) and presence (*l*_max_-limited: coloured lines) of scattering for a laser intensity of 5 × 10^13^ W cm^−^^2^. The observable manifestation of scattering is a decrease in the cut-off by Δ*E*_c_. SFA, strong-field approximation. Source data

**Extended Data Fig. 1:**
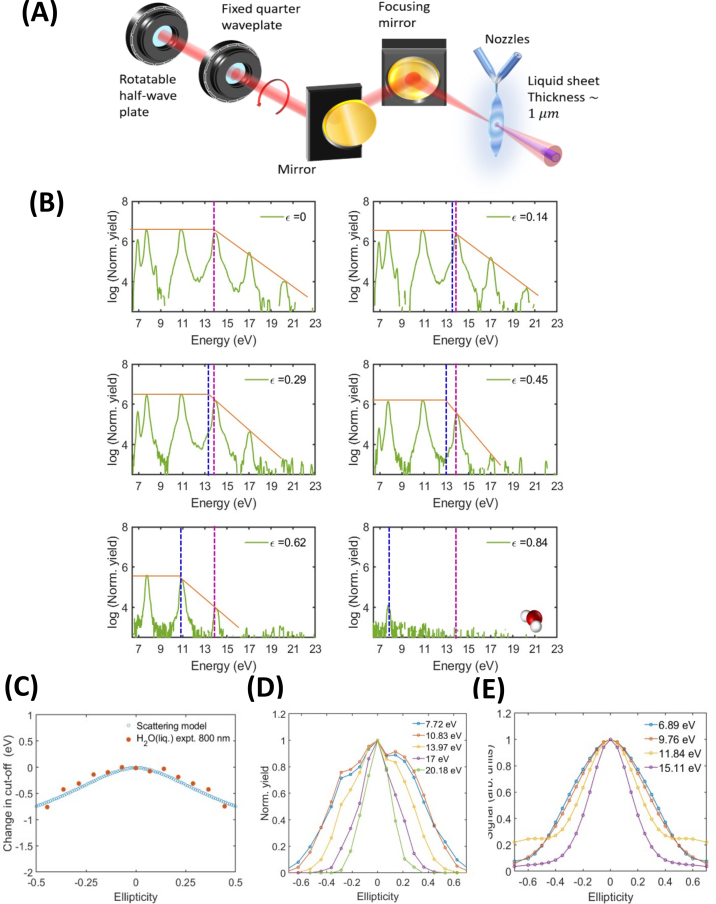
Ellipticity dependence of cutoff is consistent with mean-free-path-limited trajectory model. (A) Experimental setup for studying the ellipticity dependence of high-harmonic generation from liquids. The rotating half-wave plate and fixed quarter-wave plate geometry is used to keep the axes of the elliptically polarized driving field fixed as the light changes from linear to circular polarization. (B) Harmonic spectra obtained from liquid water at 800 nm for different driver ellipticities. The pink dashed line in each sub-figure represents the cut-off energy for linearly polarized light. The blue dashed line indicates the actual cut-off for each of these spectra. It is observed that as the light changes towards circular polarization (*ε* = 1), the cut-off shifts to lower energies. (C) Comparison of the experimentally determined change in cut-off and that calculated from the MFP-limited scattering model which considers only recombination with the parent molecule. Details of the theoretical scattering model are given in the supplementary section S3 D. (D) experimental data showing high-harmonic yields vs. ellipticity. (E) Data from cluster calculations showing the high-harmonic yields vs. ellipticity. These calculations were performed using a 900-nm driving field with a peak intensity of 4 × 10^13^W/cm^2^. Source data

One difference between HHG in dilute gases and in condensed phases is the prevalence of electron scattering in the latter. We, therefore, start our analysis by formulating a semi-classical real-space trajectory picture similar to the TSM but include scattering from the beginning. Within this picture, harmonic photons are emitted as a result of electrons following trajectories such that they recombine with their parent ion. We assume that an electron is photo-excited to the conduction band of the liquid at time *t*_ion_. Following this, the Newtonian equations of motion can be analytically solved to obtain the electron trajectory along the laser polarization axis (*x*(*t*), given in atomic units):

1 $$x(t)=\frac{q{E}_{0}}{m{\omega }^{2}}[\cos (\omega t)-\cos (\omega {t}_{{{{\rm{ion}}}}})+\omega (t-{t}_{{{{\rm{ion}}}}})\sin (\omega {t}_{{{{\rm{ion}}}}})],$$

where *E*_0_ is the peak amplitude of the laser field, *q* and *m* are the electron charge and mass, and *ω* is the angular frequency. Recombining trajectories are found by setting *x*(*t*_rec_) = 0. They have a photon energy of *Ω* = *I*_p_ + 0.5(d*x*/d*t*)^2^. The resulting cut-off is *Ω*_cutoff_ = *I*_p_ + 3.17*U*_p_, where ${U}_\mathrm{p}={E}_{0}^{2}/4{\omega }^{2}$ is the ponderomotive energy and *I*_p_ is the ionization potential. We include the effect of electron scattering by assuming that any trajectory exceeding a characteristic excursion length (denoted as *l*_max_) scatters and does not, therefore, contribute to HHG emission (Fig. 1a). The concept of trajectory clamping through electron scattering has also been used to interpret HHG in exploding plasma droplets^29^. That work, however, assumed that electron scattering can be described by neutral-molecule gas-phase scattering cross sections. Here we do not make such assumptions, but determine ${l}_{\max }$ from the experimental measurements. Note that an electron could also recombine with another centre (as reported for solids^15^). However, semi-classical calculations show that this leads to a higher-energy cut-off and a different behaviour under elliptically polarized light than measured experimentally (Extended Data Fig. 1), which allows us to discard this channel.

Typical results for the model are presented in Fig. 1c. The cut-off energy follows the TSM prediction for short wavelengths (where the trajectories do not surpass ${l}_{\max }$), but it rapidly saturates around 800 nm where the cut-off trajectories in the TSM exceed a distance of a few angstroms. As we show below, this simple model reproduces the main features of both measurements and ab-initio calculations. The non-scaling of the cut-off with wavelength is reproduced by the semi-classical model for any choice of ${l}_{\max }$, which changes only the maximal *E*_c_ (Fig. 1c). Moreover, this behaviour does not depend on the laser intensity (Supplementary Information Section 3C and Supplementary Fig. 5).

We emphasize that this simple picture likely does not capture the complete physics of strong-field light–matter interactions in the liquid phase. Nonetheless, the reproduction of the very peculiar cut-off behaviour (compared to other phases of matter) is encouraging. Also note that some of the approximations utilized here might not be accurate in the liquid phase (for example, the strong-field approximation or neglecting multicentre recombinations), but (1) corrections accounting for these effects can conceptually be added and (2) the characteristic physical behaviour of the cut-off is independent of these approximations, as demonstrated by the ab-initio results shown in TDDFT calculations section.

**Fig. 2:**
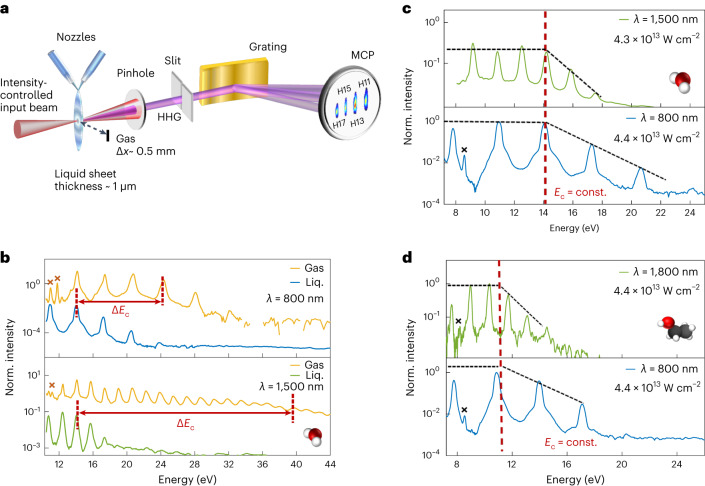
Wavelength-scaling of HHG in liquids and gases. **a**, Schematic of the experimental set-up. Laser pulses with central wavelengths of 800, 1,500 or 1,800 nm are focused onto a flat liquid jet to generate high harmonics. The generated high harmonics pass through a slit into the extreme-ultraviolet spectrometer that disperses and records the different harmonic orders. **b**, High-harmonic spectra from liquid and gaseous water recorded under identical conditions using an 800 nm (top) or 1,500 nm (bottom) driver. The difference in the cut-off is indicated by the red arrows. The normalized liquid spectra were divided by a factor of 500 (at 800 nm) and 1,000 (at 1,500 nm) for better visualization. **c**, High-harmonic spectra from liquid water recorded at different wavelengths but very similar intensities. **d**, High-harmonic spectra from liquid ethanol recorded at different wavelengths but very similar intensities. In all panels, crosses mark the harmonics reflected in the second diffraction order of the grating. Liq., liquid; MCP, micro-channel plate detector; Norm., normalized. Source data

The experimental set-up is shown in Fig. 2a. It consists of a laser system delivering ~30–40 fs laser pulses with an adjustable central wavelength (800–1,800 nm) and a high-vacuum chamber containing the liquid flat-jet system and a flat-field imaging spectrometer. Further details are given in Methods and Supplementary Information Section 1. We measured high-harmonic spectra of water (H_2_O) and ethanol (CH_3_CH_2_OH) from the liquid and gas phases of each species at different wavelengths. Two typical background-corrected HHG spectra of water are shown in Fig. 2b. The liquid- and gas-phase spectra were recorded back-to-back to minimize drifts in the experimental parameters. Figure 2b presents HHG spectra for H_2_O with the top and bottom panels directly comparing the gas- and liquid-phase signals recorded with 800 and 1,500 nm drivers, respectively. The liquid-phase harmonics are roughly ten times brighter than the gas-phase harmonics. Both spectra exhibit a distinct plateau, followed by a sharp cut-off region where the harmonic yield drops exponentially. Notably, the cut-off energy *E*_c_ is around H9 in the liquid spectrum and H17 in the gas spectrum for 800 nm wavelength. We determined the cut-off energy following the formalism elaborated in Supplementary Information Section 2. In brief, as the harmonic yield in the cut-off region is expected to decay exponentially, the logarithmic value of the harmonic yield is fitted to a linear function of the harmonic energy. In contrast, the harmonic yield for the plateau harmonics remains constant as a function of energy. The intersection of these two lines (the linear fit of the log(yield) values as a function of the harmonic energy in the cut-off region and the line indicating the average log(yield) value of the plateau harmonics) defines the cut-off energy or the end of the plateau region. We found that the liquid phase has a much-reduced cut-off compared to the gas phase. For generation in H_2_O, the gas-to-liquid difference Δ*E*_c_ was about 10 eV at 800 nm and about 26 eV at 1,500 nm. This observation is the first hint regarding the different dominant mechanisms in each phase of matter that prevent the emission of higher-energy photons from the liquid.

We next explore the wavelength-scaling of *E*_c_. This basic property reveals information about the laser-driven electron dynamics in the liquid phase. Figure 2c,d show measured high-harmonic spectra from water and ethanol, both at two different wavelengths. All spectra display the characteristic envelope with a plateau and a sharp cut-off region. This allows us to define the cut-off energy *E*_c_ as the intersection point of the two lines that connect the plateau and the cut-off region. The details of this procedure, which is followed throughout this work, are given in Supplementary Information Section 2. For each liquid, all spectra share the same cut-off energy for the plateau, that is *E*_c_ = 14.2 eV and *E*_c_ = 11.4 eV for water and ethanol, respectively. These results substantially differ from the gas-phase results, as well as the standard TSM, which both show that for the laser intensities used, the cut-off should have extended by ~25 eV between the 800 and 1,500 nm drivers.

Notably, *E*_c_ is ~3 eV smaller in ethanol than in water. The difference in cut-off energies between these liquids is substantially larger than the difference in their bandgaps (~8 eV for H_2_O and ~8.5 eV for ethanol^34,35^). This is a crucial point, since in the gas phase, and within the standard TSM, the cut-off should vary only by the difference of these values. The larger variation indicates that the liquid structure and, more precisely, the electron dynamics in the liquid phase play an additional and yet to be specified role.

**Extended Data Fig. 2:**
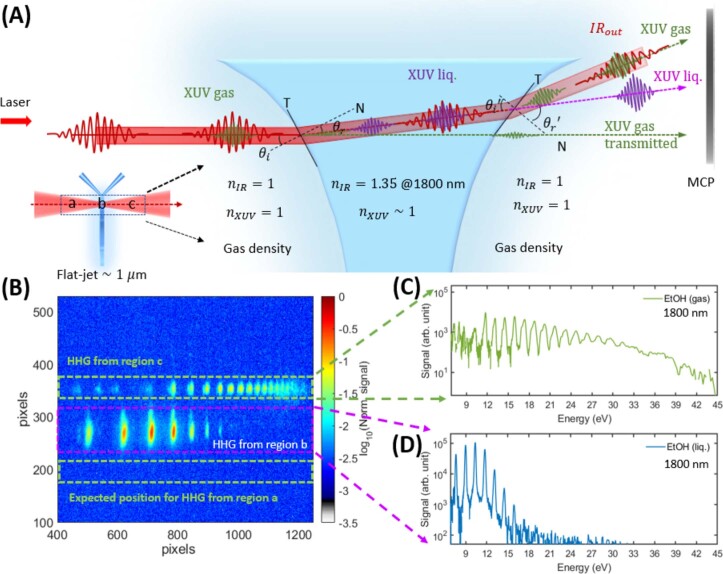
Spatially separating high-harmonic emission from the liquid and gas phases. (A) Schematic of the IR beam optical path and the high-harmonic radiation in the top part of the liquid jet (see also ref. ^26^). The bottom-left inset of panel (A) shows the overview with regions labeled a-c. The high-harmonic emission from the gas in front of the jet (region a) is absorbed in the first few layers of the liquid (~10 nm)^50^. Any XUV radiation generated in front of the liquid jet would pass unrefracted through the liquid medium. This is because the index of refraction of liquid ethanol (as well as other alcohols and water) in the relevant XUV range (7-44 eV) is very close to 1^50^. The high-harmonic radiation generated in the bulk liquid (region b) is not refracted substantially at the exit liquid-gas interface. As a result, the XUV beam is refracted negligibly in comparison to the IR beam (the refractive index *n*_IR_ is 1.35 at 1800 nm in ethanol^51^), as it exits the liquid phase into region c. In the schematic *θ*_i_ and *θ*_i_’ denote the angle of incidence of the IR beam at the entry and exit surfaces of the liquid jet. *θ*_r_ and *θ*_r_’ denote the angle of refraction of the IR beam at the entry and exit surfaces of the liquid jet and the lines T and N denote the tangent and normal to each surface. The strong refraction of the IR beam at the exit liquid-gas interface gives rise to the spatial separation of the liquid-phase harmonics (generated from the bulk liquid (region b)) and the gas-phase harmonics (generated from behind the liquid jet (region c)) of ethanol at 1800 nm, on the micro-channel plate (MCP) detector (B). The green dashed box on top of the liquid-phase harmonics indicates the region of interest on the detector selected for extracting the gas-phase harmonic spectrum (C). Similarly, the purple dashed box indicates the region of interest selected for extracting the liquid-phase harmonic spectrum (D). The green dashed box below the liquid-phase harmonics indicates the region where the transmitted gas-phase XUV emission (generated in front of the liquid jet (a)) is expected to be located on the detector. The absence of these harmonics is explained by their expected absorption in the liquid jet. Source data

**Extended Data Fig. 3:**
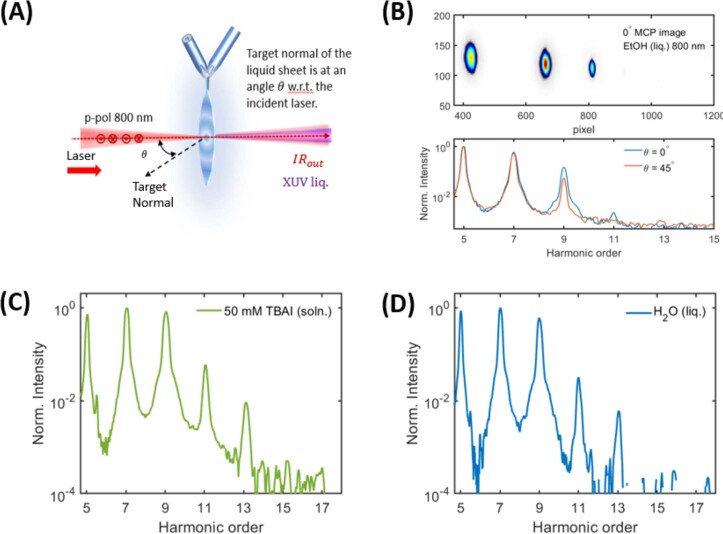
Demonstration of the dominance of bulk over surface emission of HHG through the absence of even-order harmonics. (A) Schematic showing the flat-jet orientation with respect to the incident laser propagation direction used for excluding the presence of surface/interface-generated harmonics. The p-polarized (perpendicular to the plane of the figure) 800-nm laser beam is made incident on the flat-jet. The flat-jet is rotated such that its target normal is oriented at an angle *θ* with respect to the incident laser direction. If harmonics are generated at the interface where the beam exits the flat-jet, the liquid medium would break the symmetry of the light field (and therefore the electron trajectories) between each consecutive half cycles of the electric field for any *θ* > 0, which would lead to the generation of even harmonics. (B) The raw MCP image for *θ* = 0° (top panel) and the comparison of the harmonic spectra (bottom panel) taken for *θ* = 0° (blue line) and *θ* = 45° (orange line), for 800 nm laser beam incident on an ethanol flat-jet. No even harmonics are detected for the tilted jet geometry, which suggests that the detected signals are dominated by the bulk liquid with negligible interface contributions. (C) We also studied the harmonic emission from 50-mmol Tetrabutylammonium iodide (TBAI) solution at 45° jet orientation. TBAI was chosen here because of its high surface affinity caused by the hydrophobic interactions of the TBA^+^ cation and the large polarizability of the I^−^ anion^52^. This should further enhance the visibility of any even surface/interface harmonics arising from the breaking of the inversion symmetry of the p-polarized driver by the liquid jet. We also compared this spectrum with that of pure water (D) for the same 45° jet orientation. However, even harmonics were not observed in any of these cases, indicating that the surface/interface contribution to the HHG response is negligible as compared to that of the liquid bulk. Source data

A critical aspect of measuring HHG in liquids is to ensure that the measured signals originate from the bulk liquid phase. This requires explicitly excluding HHG emission from the evaporating gas phase as well as HHG from the gas–liquid interface. A complete experimental separation of HHG emission from the gas and liquid phases has been achieved by using the wedge-like geometry in the upper part of the liquid jet, as shown in Extended Data Fig. 2. Additional experiments with the liquid jet placed at an angle of 45° with respect to the driver-beam propagation direction allowed us to exclude contributions from HHG at the liquid–gas interface because of the absence of any measurable even harmonics (Extended Data Fig. 3).

**Extended Data Fig. 4:**
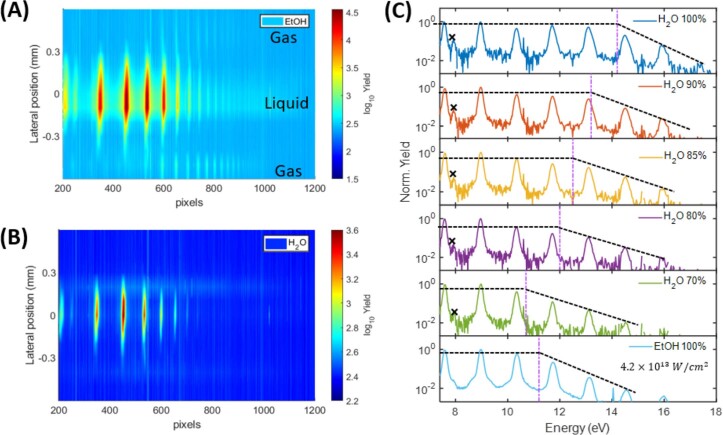
High-harmonic generation in liquid water-ethanol mixtures. (A) Lateral scans of ethanol at 1800 nm: Raw HHG spectrum of pure EtOH at 4.2 × 10^13^ W/cm^2^ peak intensity as a function of the lateral jet position, where x=0 indicates the laser beam hitting the center of the flat jet. It can be seen that the horizontal width of the liquid jet is 0.6 mm, outside of which we only observe the much weaker gas-phase harmonics. (B) Lateral scans of H_2_O at 1800 nm: Raw HHG spectrum of pure H_2_O at 4.2x10^13^ W/cm^2^ peak intensity as a function of the lateral jet position. Here we do not observe any gas-phase signal. (C) Comparison of the high-harmonic spectrum of pure liquid H_2_O, H_2_O:EtOH solvent mixtures and pure liquid EtOH at 1800 nm in the ratio of 90:10, 85:15, 80:20, 70:30 and pure EtOH at x=0 for 4.2 × 10^13^ W/cm^2^ intensity. For comparison, each spectrum is normalized to the harmonic at 8.9 eV. In all panels, crosses mark the harmonics reflected in the second diffraction order of the grating. The black dashed lines are guides for the eye while the magenta dashed lines indicate the cut-off energies calculated using the algorithm explained in section 2 of SM. All data in this figure have been acquired for 600 ms and averaged over 30 scans at an MCP voltage of -1.7 kV and phosphor voltage of 3.3 kV. Source data

We, thus, reached two main conclusions: (1) the position of the cut-off in the liquid-generated high-harmonic spectra depends on the nature of the liquid sample (Extended Data Fig. 4) and (2) the cut-off energy is wavelength-independent, at least in water and ethanol. In what follows, we will show that these results are reproduced by ab-initio calculations.

**Fig. 3:**
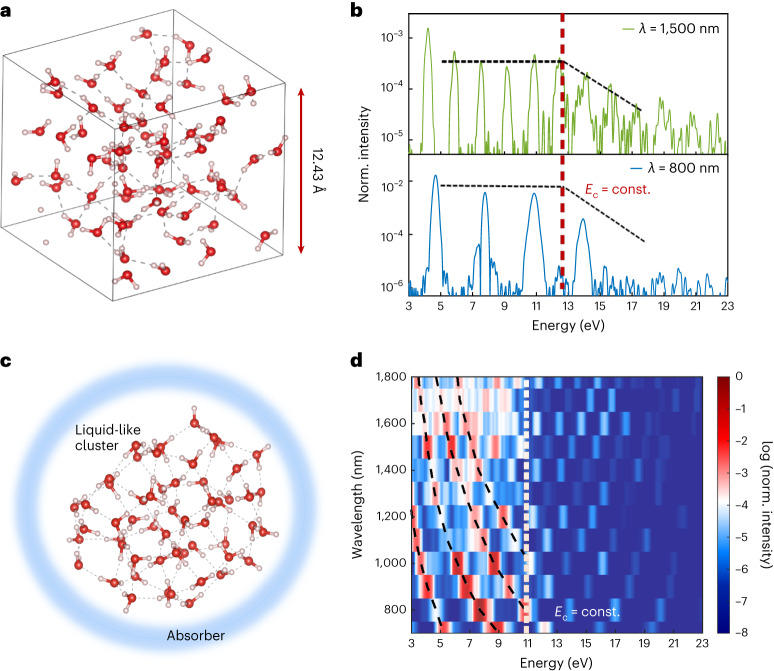
TDDFT calculations. **a**, Schematic representation of liquid water in the supercell approach. There are 64 water molecules in a cubic cell at the experimental density of 1 g cm^−^^3^. Periodic boundary conditions were implemented in three dimensions. Each lattice vector was of length ~12.43 Å. **b**, HHG spectra calculated for liquid water using two different driving wavelengths. The peak intensity for all wavelengths was the same and was equal to approximately 20 TW cm^−^^2^. These HHG spectra are averaged over 5,000–5,500 water molecules in the liquid phase (for details, see the Supplementary Information and ref. ^38^). **c**, Schematic representation of liquid water in the cluster approach with a cluster radius of ~15.5 Å. **d**, Wavelength-scaling of high-harmonic spectra calculated for a constant peak intensity. Norm., normalized. Source data

**Extended Data Fig. 5:**
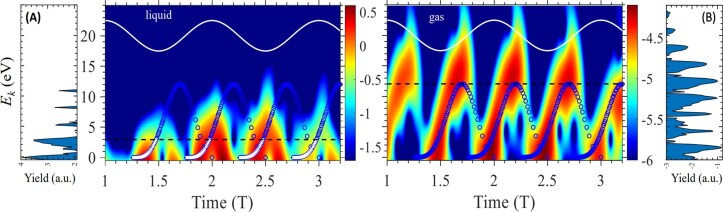
Time-frequency analysis of HHG in clusters supports mean-free-path-limited trajectory model. (A) Time-frequency analysis for calculated HHG emission from the liquid cluster, comparing the ab-initio results to the simple trajectory-based model that includes MFP-limited trajectories. The y-axis shows the emitted HHG photon energy relative to the HOMO-LUMO (lowest unoccupied molecular orbital) gap in the cluster, which corresponds to the kinetic energy of the electrons upon recombination. The x-axis is given in units of an optical cycle. The gas-phase expected SFA trajectories are shown as blue circles, while the remaining short trajectories that do not exceed the MFP are highlighted with white crosses. The corresponding HHG spectra are plotted for reference, along with the laser electric field (in solid white). The cutoff from the semi-classical model is indicated in dashed black. (B) Same as (A) but for an isolated molecule, where the response is orientation averaged to describe an un-oriented gas of water molecules. The cutoff in dashed black indicates the one obtained from SFA trajectories, and only SFA trajectories from the highest occupied molecular orbital (HOMO) orbital are plotted (while some low-yield higher-energy emission still exists due to emission from the HOMO-1 and HOMO-2 orbitals). Both plots share the same kinetic-energy scale and are calculated for 900-nm laser driving with an intensity of 5 × 10^13^ W/cm^2^. The plot clearly indicates that in the liquid phase there is a dominant very-short trajectory contribution, whereas longer trajectories are strongly suppressed. The ab-initio cutoff for the liquid agrees very well with the prediction from the semi-classical model that incorporates the MFP as extracted from experiments. The time-frequency structure for the gas-phase similarly agrees with the expected structure, validating the approach. We note an additional emission contribution in the liquid phase at longer times that possibly corresponds to some other scattering process or a different emission channel that will be investigated in future work. The time-frequency analysis is obtained with a Gabor transform with a Gaussian window of T/3, and the isolated molecule calculations are obtained with the methodology described in ref. ^39^. Source data

We now compare these experimental findings and the results of our simple model to two newly developed ab-initio techniques for describing the strong-field light–matter response of liquids. Figure 3 presents simulated HHG spectra from liquid water that are based on a combination of well-established Car–Parrinello molecular dynamics^36^ and time-dependent density functional theory (TDDFT)^37^ simulations in a periodic supercell with 64 water molecules at the experimental density of 1 g cm^−^^3^ and temperature of 300 K (for details, see ref. ^38^ and Supplementary Information Section 3.A). This is a realistic and currently tractable description of HHG in liquids. TDDFT naturally includes effects due to the mean free path (MFP) as it includes electron–electron and electron–ion scattering. Overall, a very good agreement with the experimental results was observed, and most importantly, the cut-off energy and its wavelength-independence are well reproduced in Fig. 3. Moreover, a time–frequency analysis of the TDDFT results (Extended Data Fig. 5) shows that only very short electron trajectories contribute to the HHG spectra, in agreement with our semi-classical model. Note that since the generalized gradient approximation in density functional theory underestimates the liquid water bandgap, the calculated HHG cut-off is about 1.5 eV lower than the experimental value. This numerical approach qualitatively reproduces the experimentally observed weak dependence of the cut-off on the laser intensity (see the discussion in Supplementary Information Section 3.A). This further confirms that the above experimental findings are a signature of the microscopic mechanism in the liquid phase and not the result of macroscopic effects, which are absent in our theoretical modelling.

This result is complemented by a second set of ab-initio TDDFT calculations based on molecular clusters that employ some additional approximations (for details, see ref. ^39^). The advantage of this approach is that the calculations are faster while still producing accurate results; thus, it can be employed for a more detailed numerical study. Figure 3d shows simulation results for HHG in liquid water with the cluster approach for many wavelengths at a fixed laser intensity. Clearly, the same trend is observed and the cut-off is independent of the wavelength, at least in the range 500–1,800 nm. In Supplementary Information Section 3.B, we show that the cut-off with the cluster approach is similarly weakly dependent on the laser intensity (above a saturation intensity of 5 × 10^13^ W cm^−^^2^) and that the wavelength-independence of the cut-off is maintained for other laser intensities, as well. With the cluster approach, we also performed calculations for two additional liquids (ammonia (NH_3_), which is polar, and methane (CH_4_), which is non-polar). In Supplementary Information Section 3.B, we show that HHG calculations for liquid NH_3_ and liquid CH_4_ also predict the same wavelength-independence of *E*_c_. These results, in combination with our measurements, lead to the conclusion that this characteristic non-scaling of *E*_c_ is a fundamental, general and unique property of the liquid-phase HHG and applies for both polar and non-polar liquids. These accurate quantum-dynamical simulations reproduce and complement our experimental findings, which validates the broad applicability of our conclusions.

**Fig. 4:**
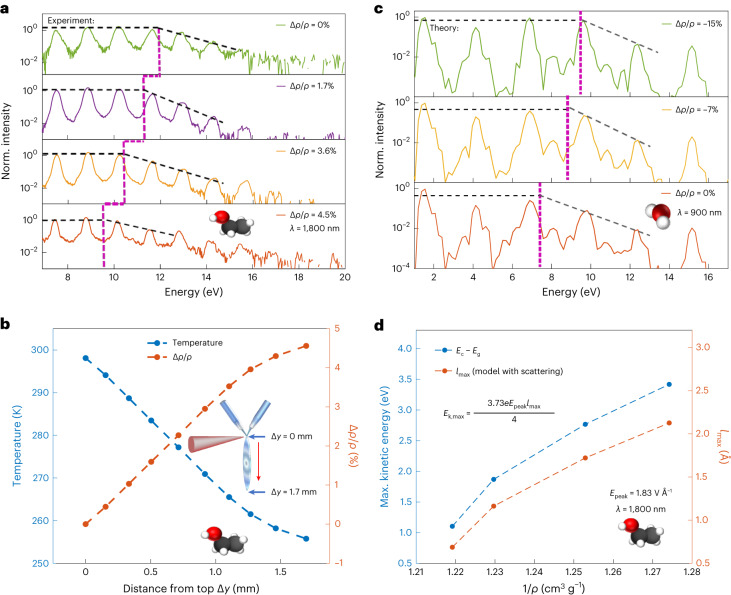
Scaling of the cut-off energy with the density of the liquid. **a**, Measured high-harmonic spectra (1,800 nm driver, 6 × 10^13^ W cm^−^^2^) as a function of vertical position on the liquid flat-jet, corresponding to the indicated change in density. The change in density was determined from an absolute temperature measurement carried out by Raman thermometry^40^, as shown in **b**. **c**, Spectra calculated with the ab-initio cluster approach (900 nm, 4 × 10^13^ W cm^−^^2^) for varying densities (Δ*ρ*/*ρ* = −15%, −7% and 0%). The harmonics above the cut-off show a systematic decrease in yield with increasing density. **d**, Maximal observed kinetic energy as a function of the inverse density for ethanol and the corresponding ${l}_{\max }$ obtained from the formula shown in the inset (derived in Supplementary Information Section 3.D). The vacuum electric field was corrected using the 1,800 nm refractive index in ethanol to obtain the peak electric field (*E*_peak_) inside the liquid. Norm., normalized. Source data

We have so far demonstrated the wavelength-independence of *E*_c_, both experimentally and theoretically (Figs. 2 and 3), and we have shown that a scattering-limited trajectory model reproduces this behaviour (Fig. 1). Importantly, a main conclusion arising from our results is that if *E*_c_ is limited by the effective electron MFP *λ*_MFP_ in the liquid, then *E*_c_ should scale with the density of the liquid. Figure 4 demonstrates that this is the case, both experimentally (Fig. 4a) and theoretically (Fig. 4c).

Very recently, some of us have reported measurements of the temperature of liquid flat-jets^40^. The temperatures, measured by Raman thermometry under conditions identical to those of the present HHG experiments, range from ~300 K at the top to ~255 K at the bottom of the first sheet, translating to a density variation of close to 5% (Fig. 4b) for ethanol^41^. Over this range of conditions, *E*_c_ decreased by ~2 eV (Fig. 4a). The same trend was also observed in the calculations performed for liquid water (Fig. 4c). Having measured *E*_c_ over a range of densities, we can now verify how the maximal energy *E*_k,max_ gained by the electron from the driving laser field scales with the density. Experimentally, we use ${E}_{{\mathrm{k}},{\mathrm{max }}}={E}_{\mathrm{c}}-{E}_{\mathrm{g}}$, where *E*_g_ is the bandgap of the liquid. We find that *E*_k,max_ scales linearly with the inverse density (blue symbols in Fig. 4d). This type of scaling precisely corresponds to the prediction of our simple trajectory-limited model (Fig. 1), because *λ*_MFP_ = 1/(*nσ*) ∝ 1/(*ρσ*), where *n* is the number density of the molecules and σ is the scattering cross-section.

This conclusion is further supported by converting the measured *E*_c_ to the corresponding maximal excursion length ${l}_{\max }$. As we show in Supplementary Information Section 3.D, we find that ${E}_{{\mathrm{k}},{\mathrm{max }}}=(3.73/4)eE{l}_{\max }$. A direct consequence of this relation is that it allows us to retrieve ${l}_{\max }$ from the experimental spectra, provided that they were recorded under conditions where the wavelength-independence of the cut-off is observed, which is the case here (Fig. 2c,d). The orange symbols in Fig. 4d show that ${l}_{\max }$ also scales linearly with the inverse density.

We, therefore, conclude that all the experimental and theoretical evidence available at present agrees with stating that *E*_c_ is proportional to the maximal excursion length of the laser-driven electrons in the liquid phase. This suggests that it should be possible to accurately determine effective electron MFPs (*λ*_MFP_) from liquid-phase HHG spectra.

**Fig. 5:**
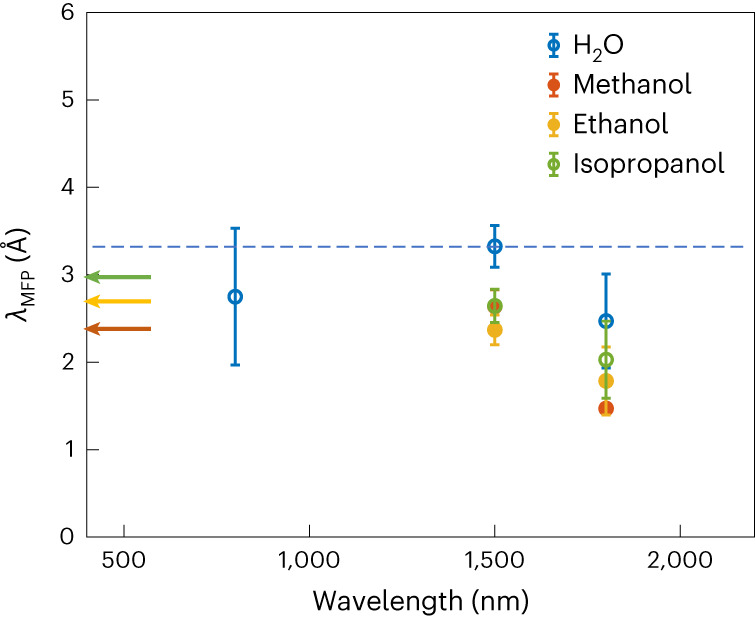
Comparison of the determined effective electron MFPs with literature data. MFPs for electron scattering in the liquid phase determined from the experimentally observed cut-off energies (*E*_c_) as a function of laser wavelength. In the semi-classical formula, the MFP depends on the electric-field amplitude. The error bars around each experimental data point represent the minimum and maximum MFPs calculated for a range of intensities for each liquid. The lower limit of each error range corresponds to the MFP calculated from the semi-classical formula for the maximum intensity and the higher limit corresponds to the MFP for the minimum incident intensity for HHG in the specified liquid. The points represent the mean values. The dashed blue line indicates the elastic MFP for liquid water^43^ at *E*_k_ = 4.5 eV. The arrows indicate the MFPs obtained from the integral elastic scattering cross sections of the corresponding alcohols in the gas phase^47,48^ for an *E*_k_ of ~3–4.5 eV, using the number densities obtained from the densities of different liquids at 20 °C (ref. ^41^). Source data

Electron MFPs play a very important role in describing electron-driven processes in the liquid phase^42^, but they are notoriously difficult to measure and calculate, especially at low energies. The interest in developing new methods for accessing these quantities is, therefore, considerable and relevant for many physical and chemical processes. Here we do not attempt to determine the MFPs with high precision because this would require a more sophisticated scattering model, including a large number of different scattering channels (see ref. ^43^ and references therein). Instead, we aim at retrieving an effective MFP (*λ*_MFP_), which is best thought of as accounting for all types of scattering processes. Since the elastic scattering cross sections are by far dominant at the very low kinetic energies (~10 eV) of interest here^43–46^, we compare our results to the elastic MFPs in Fig. 5. In this comparison, we use ${\lambda }_{{{{\rm{MFP}}}}}={l}_{\max }$, taking into account that the electron travels up to the maximal excursion length before being scattered. In Supplementary Fig. 9, we show that this simple approximation is physically meaningful because replacing the ‘sharp’ truncation of the trajectories (at the travel distance ${l}_{\max }$) with an exponential distribution of path lengths (inherent to the definition of *λ*_MFP_) leaves *E*_c_ unchanged. Figure 5 compares the *λ*_MFP_ values obtained from the HHG spectra (symbols) with the available literature values. For liquid water, we are comparing the values to the most recent MFPs (blue dashed line), which were determined from a Monte Carlo simulation of experimental liquid-microjet data using the most accurate ab-initio differential scattering cross sections available to date^43^. For the alcohols, liquid-phase MFPs have, to our knowledge, not been reported in the literature so far. We are, therefore, comparing our results to MFPs determined from the corresponding experimental gas-phase elastic scattering cross sections and the known number densities of the alcohols. The agreement is very good in all cases, confirming the possibility of retrieving effective electron MFPs from liquid-phase high-harmonic spectroscopy. The remaining uncertainties in the retrieved *λ*_MFP_ originate from the determination of *E*_c_ (Supplementary Fig. 1), the exact value of the bandgap and the limitations of our simple model, which retrieves a single parameter (*λ*_MFP_) and neglects its energy dependence over the small range of kinetic energies (*E*_k_ ≤ 4.5 eV) that are accessed in the present experiments. Improved scattering models^43,46^ and refined retrieval algorithms will alleviate these limitations.

To summarize, we explored here the microscopic mechanisms responsible for liquid-phase HHG with a combination of experimental and theoretical methods. Our measurements of water and ethanol show that, contrary to crystals and gases, the cut-off energy in liquid-phase HHG is mostly independent of the laser wavelength. Microscopic quantum mechanical calculations based on both supercells and clusters agree with this result and show that it extends to other liquids and laser conditions. We showed that an extended semi-classical model that incorporates the effects of the ultrafast scattering of electrons off neighbouring molecules is capable of explaining the reduced HHG cut-off of liquids compared to the gas phase. The model reproduces well the wavelength-independence of the HHG cut-off and highlights the importance of the electron MFP in liquids, indicating that this quantity is imprinted onto the high-harmonic spectra and can be retrieved. We also expect that our results are highly relevant for HHG from amorphous solids^25,26^. Our work may pave the way to a deeper understanding of the strong-field dynamics in disordered condensed phases and to resolving attosecond dynamics in liquids.

## Methods

The experimental set-up consists a 1 kHz Ti:sapphire laser delivering ~30 fs pulses at 800 nm. Driving wavelengths of 1,500 and 1,800 nm were obtained by optical parametric amplification of the 800 nm pulses. The driver beams were focused by a spherical mirror onto a micrometre-thin liquid flat-jet target, as further described in refs. ^30,31^. The beam intensities were calculated from the harmonic cut-off energy of the gas-phase measurements, using the semi-classical TSM^1^. The emerging high harmonics were analysed with a custom-built extreme ultraviolet spectrometer consisting of an aberration-free flat-field grating (Shimadzu, Japan) and a multichannel-plate detector coupled with a phosphor screen. The image on the phosphor screen was recorded by a charge-coupled-device camera. Each spectrum was typically integrated over 10–20 ms and 200 ms, for the gas and liquid phases, respectively, and measured 20 times. These spectra were averaged before all subsequent analysis steps. For the ellipticity-dependent studies, elliptically polarized 800 nm pulses were generated using a combination of a rotating half-wave plate and a fixed quarter-wave plate. The rotation of the half-wave-plate axis from 0° to 22.5° with respect to the quarter-wave-plate axis changes the polarization of the input light from linear to circular while keeping the axes of the polarization ellipse fixed. At a number of different ellipticities, ranging from *ϵ* = 0 for linear polarization to *ϵ* = 1, for circular polarization, the harmonic spectrum was measured for different liquids. Further details of the experimental methods are described in Supplementary Information Section 1 and Extended Data Figs. 1 and 2.

### Intensity scaling of gas- and liquid-phase HHG spectra

**Extended Data Fig. 6:**
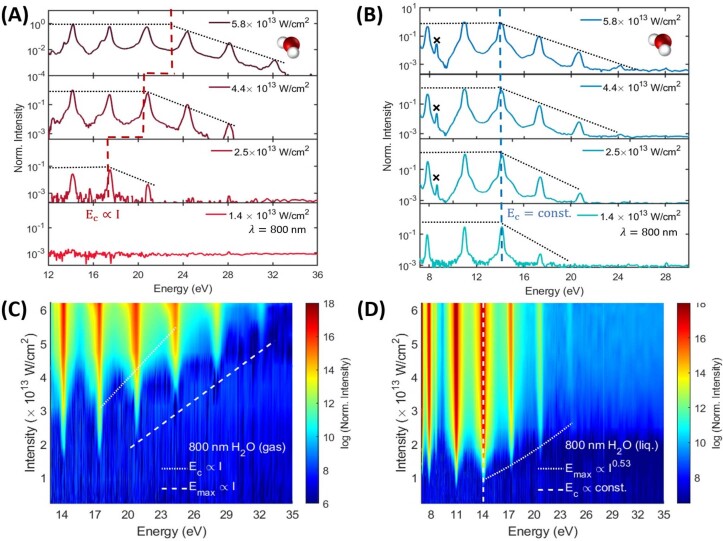
Comparison of gas- and liquid-phase HHG spectra and their intensity scalings. For 800-nm driver, a comparison between gas- (A) and liquid- (B) phase high-harmonic spectra of H_2_O is given for four intensities. As expected, the gas-phase cut-off energy depends on intensity (red dashed line) but the liquid-phase cut-off energy is nearly intensity independent (blue dashed line). For 800 nm driver, a comparison between gas- (C) and liquid- (D) phase high-harmonic spectra of H_2_O is given for a range of intensities. Source data

Extended Data Fig. 6 presents the experimentally measured HHG spectra emitted from liquid water at an 800 nm driving wavelength for different laser intensities in the range 1.4 × 10^13^ to 5.8 × 10^13^ W cm^−^^2^. At each intensity, we compared the gas-phase (Extended Data Fig. 6a) and liquid-phase (Extended Data Fig. 6b) spectra. For the gas phase, there was a systematic linear increase of the cut-off with laser intensity (red dashed line), in accordance with the standard TSM. In contrast, the liquid HHG cut-off energy remained roughly constant (blue dashed line). This effectively demonstrates that in the given intensity range, the cut-off energy of the liquid spectra is independent (or weakly dependent) on the peak laser intensity. We also verified that this is the case for liquid ethanol.

### Scaling of the highest harmonic order versus scaling of the plateau cut-off

When relating the present results to those in the literature, it is important to distinguish the scaling of the highest emitted harmonic order (${E}_{\max }$) from the scaling of the plateau cut-off (denoted *E*_c_, and discussed in the main text). Previous works on condensed-phase HHG have mainly studied and discussed the scaling of ${E}_{\max }$. Most prominently, this is the case for Ghimire et al. for the solid state^8^ and for Luu et al. for the liquid phase^30^. In contrast, the overwhelming majority of the literature on gas-phase HHG has studied and discussed the scaling of *E*_c_, being influenced by the definition provided by Lewenstein et al.^2^. Here we show that the scaling of these two quantities is actually different in the liquid phase and thereby show that the present conclusion regarding the scalings of *E*_c_ are consistent with previous results in the literature, in particular those of Luu et al.^30^.

Extended Data Fig. 6c,d shows the data in a form that highlights the scalings of both ${E}_{\max }$ and *E*_c_. Extended Data Fig. 6c shows that, in the gas phase, both ${E}_{\max }$ and *E*_c_ scale linearly with the intensity. Extended Data Fig. 6d, in contrast, shows that the scalings of the two quantities are noticeably different in the liquid phase. A nonlinear least-squares fitting of ${E}_{\max }$ to the functional form ${E}_{\max }\propto {I}^{\,y}$ returns *y* = 0.53, consistent with Luu et al.^30^. The independence of *E*_c_ on *I* is visible, both in Extended Data Fig. 6b and in Extended Data Fig. 6d. This validates the conclusions reached in the main text and clarifies their relation with previous work.

We also performed ab-initio calculations to test the intensity dependence of the cut-off with both the supercell (Supplementary Fig. 2) and the cluster (Supplementary Fig. 4) approach for liquid HHG. Both types of calculations show that the cut-off is independent of the laser driving intensity, beyond a certain threshold. In each case, the cut-off increases with the laser intensity until it reaches a saturation point, where the increase stops. For the supercell approach, this saturation was at ~0.25 × 10^14^ W cm^−^^2^, whereas for the cluster approach, it was at ~0.5 × 10^14^ W cm^−^^2^. The differences between the results for the two methods are due to the slightly different electronic structures (bandgaps, in particular) obtained with the two approaches. Notably, the ranges of laser intensities for which the cut-off is intensity independent correspond well with the experimental measurements.

Lastly, we also point out that the observed weak dependence of the HHG cut-off with respect to the laser intensity is also captured by our proposed extended semi-classical model. Supplementary Fig. 5 presents the calculated HHG cut-off versus the peak laser field with the extended semi-classical model, assuming 1500 nm driving and intermolecular distances like those in liquid water. Initially, the HHG cut-off increases quadratically with the laser intensity just as in the standard TSM (because the trajectories are very short and do not extend beyond *l*_max_). However, this dependence is reduced to a weak linear scaling in the range of intensities >0.5 × 10^14^ W cm^−^^2^. In fact, over the intensity range 0.5 × 10^14^ to 10^14^ W cm^−^^2^, the HHG cut-off increases by only ~2 eV. This result substantially differs from the gas phase, for which the standard TSM predicts that the cut-off should increase by ~9 eV in that region. Notably, a change of ~2 eV in the cut-off energy at 1500 nm driving would move the HHG cut-off only by approximately one odd harmonic order, which might be difficult to detect experimentally. Thus, we conclude overall that in our examined conditions, the liquid HHG cut-off is weakly dependent on the laser driving intensity, an effect that is described remarkably well by our suggested semi-classical picture that includes scattering. We note that potential improvements to our extended semi-classical model (for example, relaxing some of the approximations utilized) might also improve its correspondence to the measured and calculated (ab-initio) results.

## Online content

Any methods, additional references, Nature Portfolio reporting summaries, source data, extended data, supplementary information, acknowledgements, peer review information; details of author contributions and competing interests; and statements of data and code availability are available at 10.1038/s41567-023-02214-0.

### Supplementary information

Supplementary Information Supplementary Figs. 1–15 and discussion.

### Source data

Source Data Fig. 1 Numerical data for the calculated results shown in Fig. 1.

Source Data Fig. 2 Numerical data for the high-harmonic spectra shown in Fig. 2.

Source Data Fig. 3 Numerical data for the high-harmonic spectra shown in Fig. 3.

Source Data Fig. 4 Numerical data for the results shown in Fig. 4.

Source Data Fig. 5 Numerical data for the results shown in Fig. 5.

Source Data Extended Data Fig. 1 Numerical data for the results shown in Extended Data Fig. 1.

Source Data Extended Data Fig. 2 Numerical data for the results shown in Extended Data Fig. 2.

Source Data Extended Data Fig. 3 Numerical data for the results shown in Extended Data Fig. 3.

Source Data Extended Data Fig. 4 Numerical data for the results shown in Extended Data Fig. 4.

Source Data Extended Data Fig. 5 Numerical data for the results shown in Extended Data Fig. 5.

Source Data Extended Data Fig. 6 Numerical data for the results shown in Extended Data Fig. 6.

## Data Availability

The datasets generated during and/or analysed during the current study are available from the corresponding author on reasonable request. The data for the figures presented in this manuscript are available from 10.3929/ethz-b-000595173 (ref. ^49^). Source data are provided with this paper.

## Extended data

Extended data are available for this paper at 10.1038/s41567-023-02214-0.

## Code availability

The Octopus package used for the TDDFT calculations is publicly available. The remaining computer codes are available from the corresponding author on reasonable request.
