# Supplementary material for: High-harmonic spectroscopy of low-energy electron-scattering dynamics in liquids
Source: Nat Phys. 2023 Sep 28;19(12):1813–20. doi: 10.1038/s41567-023-02214-0 (PMC10709138; doi:10.1038/s41567-023-02214-0)
Supplement: Supplementary file 1 — Supplementary Figs. 1–15 and discussion. [file 41567_2023_2214_MOESM1_ESM.pdf]

nature physics

Article

https://doi.org/10.1038/s41567-023-02214-0

High-harmonic spectroscopy of low-energy electron-scattering dynamics in liquids

In the format provided by the authors and unedited

CONTENTS

S1. Experimental details 1
A. Optical beamline 1
B. Flat Jet 1

S2. Determination of harmonic cut-off energy from experimental data 1

S3. Theoretical details 2
A. ***Ab initio*** supercell calculations 2
1. Molecular dynamics simulations 2
2. Ultrafast dynamics simulations 2
3. Pulse characteristics 2
B. Cluster approach ab-initio calculations 3
C. Extended semi-classical calculations 4
D. Semi-classical description of HHG and cutoff scaling including scattering 4

S4. Intensity dependence of liquid-HHG cutoff 5

S5. Comparative study of cut-off energy with different trajectory-truncation methods 6

S6. Analytical treatment of the propagation effect 7
A. Measured intensity for thick films 10
B. Measured intensity for thin films 10
C. Results for the propagation effects 11

S7. Experimental results for density and propagation effects 11

S8. Comparison of cut-off energy obtained from the semi-classical scattering model from nearest neighbor interaction and onsite interaction 13

S9. Change of the cutoff energy for elliptically polarized light 13

S10. Comparison with a prior work on HHG from liquid droplets 14

References 15

S1. EXPERIMENTAL DETAILS

A. Optical beamline

The experiments were performed with a 1 kHz regeneratively-amplified Ti:Sapphire laser (Coherent). From the laser output, 1.2 mJ beam was split and directed to a dedicated grating compressor delivering about 960 μJ, $\sim$30 fs pulses with a central wavelength of 800 nm. The linear and vertically (s) polarized driving beam was focused on a liquid flat-jet target with a pair of metallic mirrors (protected Ag) in a Z-shaped configuration, where the first mirror was flat and the second mirror was spherical with R = 800 mm. The whole set was mounted on a common base plate on top of a manual translational stage to allow fine tuning of the overlap between the laser focus and the flat jet.

For experiments with mid-IR wavelengths (1500 nm and 1800 nm), a commercial optical parametric amplifier (OPA, HE-TOPAS from Light Conversion) pumped with 6.5 mJ, 30 fs pulses at 800 nm was used. The signal or idler beam was separated from the residual wavelengths exiting the OPA using a pair of dielectric mirrors for the particular wavelength and focused with the same set of two metallic mirrors.

B. Flat Jet

The flat jet was created by two colliding cylindrical jets with about $\sim$54 μm inner diameter. The samples were pumped via an HPLC pump (JASCO) into the interaction region with a flow rate ranging from 3 ml/min to 5 ml/min. The established flat jet was about 1 μm thick [1]. Due to evaporation, the liquid jet was surrounded by a region with high gas density, as compared to the backing pressure in the target chamber ($\sim 5 \cdot 10^{-4}$ mbar), allowing a simultaneous probing of liquid and gas phases. All liquid samples have been used without further purification: pure Milli Q water with an electric resistivity of 18 MΩ·cm and 99.8 % pure alcohols (ethanol, methanol, iso-propanol) from Sigma Aldrich.

S2. DETERMINATION OF HARMONIC CUT-OFF ENERGY FROM EXPERIMENTAL DATA

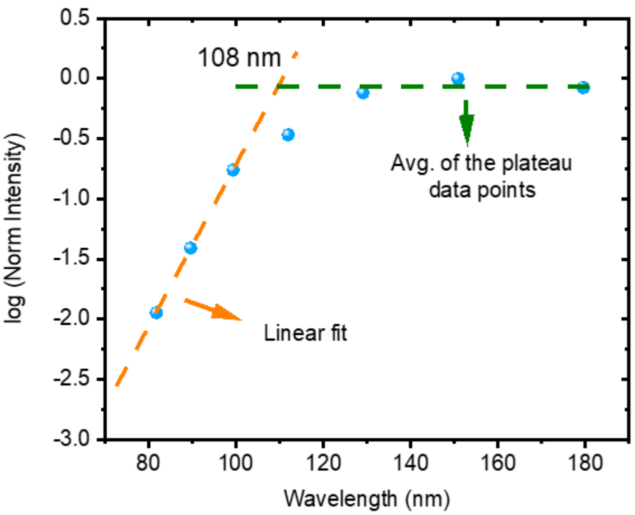

FIG. S1. Plot of harmonic signal as a function of harmonic wavelength. The cut-off is determined by the intersection of the green and orange lines.

In order to locate the cut-off from the experimental

spectra we use the following method. The signal at each harmonic wavelength from the experimental spectra is acquired. It is known that the harmonic signal beyond the plateau region shows as exponential decay, which results in a linear decline in the logarithmic scale. Fig. S1 depicts this clearly, where the plateau region has a constant harmonic yield followed by a linear decay in the harmonic signal. The green line indicates the average signal of the plateau harmonics and the orange line indicates a linear fit to the intensities in the cut-off region. The intersection of the green and orange lines denotes the cut-off wavelength, which is converted to E_c, the cut-off energy of the plateau.

S3. THEORETICAL DETAILS

A. *Ab initio* supercell calculations

1. *Molecular dynamics simulations*

In order to study theoretically the interaction of strong-field pulse with liquid water, the first step is simulating the structural and dynamical properties of liquid water. In this regard, we use Car-Parrinello molecular dynamics (CPMD) simulations in the canonical ensemble at the temperature of 300 K. The simulated system, as displayed in the main text, is a periodic cubic supercell, including 64 H_2O molecules in the experimental liquid water density of 1 g/cm^2. Our computed radial distribution function between oxygen atoms (shown in Ref. [2]), which is averaged over a time length of approximately 20 ps, is in a good consistent with the experimental neutron scattering and x-ray diffraction measurements; it confirms the accuracy of our liquid water molecular dynamics simulation. Our CPMD simulations are performed using Quantum Espresso package [3]. We use norm-conserving GGA-revPBE [4] pseudopotentials for oxygen and hydrogen atoms and Grimme-D2 dispersion correction to describe the non-local correlation effects. For more details please see Ref. [2].

2. *Ultrafast dynamics simulations*

In the next step, to study the interaction of ultrashort intense pulse with liquid target, we use real-time time-dependent density functional theory implemented in Octopus package [5–7]. Note that the ultrashort laser pulse stimulates the system in the time-scale of femtosecond while our CPMD simulation reproduce the macroscopic characteristics of liquid water in the time-scale of picosecond. So in order to obtain the converged response, we need to consider a sufficient number of different configurations of the liquid supercell system, denoted as 'subsystems'. The time interval between consecutive subsystems are in the order of picosecond, and the number of required subsystems for convergence depend on the target system as well as pulse characteristics (wavelength and intensity).

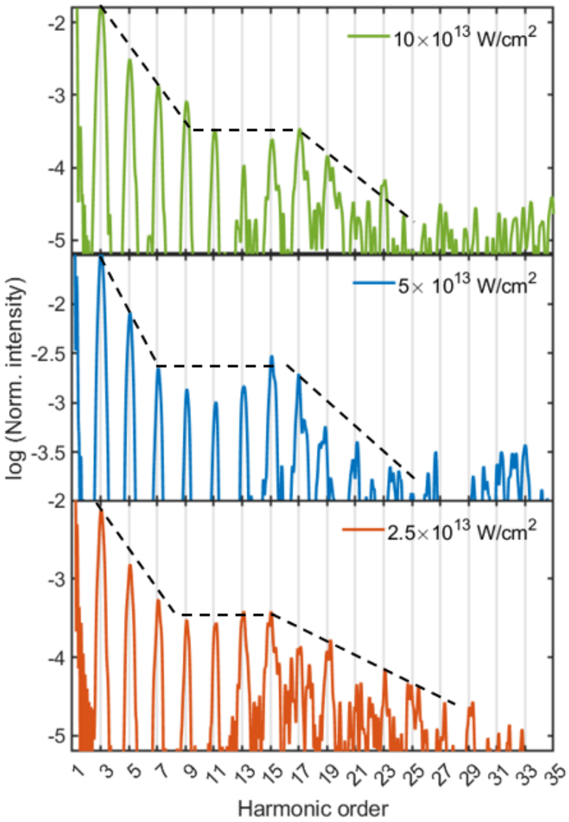

FIG. S2. High-harmonic spectra obtained from calculations with the supercell approach using a driving wavelength of 1500 nm.

In the supercell simulations, we consider the dynamics of nuclear based on Ehrenfest-TDDFT approach [8] during the interaction of ultrashort intense pulses with the liquid system. However, the effect of ionic current is negligible and the calculated HHG arises totally from electron dynamics.

The exchange-correlation term in TDDFT calculations is described by the same GGA-revPBE pseudopotentials as those used in CPMD calculations. But, vdW interactions are not included, as they lead to no difference in the spectra. We consider a grid spacing of 0.3 bohr thorough TDDFT calculations, and a dense k-point grid of $5\times5\times5$. Also, we use aetrs algorithm to approximate the evolution operator and the time step of 0.2 a.u. within our TDDFT calculations.

3. *Pulse characteristics*

In order to evaluate the impact of pulse wavelength and its intensity on HHG spectra, we consider the pulse

characteristics corresponding to our experimental conditions. To see the impact of driving wavelength on HHG cutoff energy, we consider two different wavelengths of 800 and 1500 nm under the constant intensity of about 20 TW/cm^2 (shown in Fig. 3B of the main text). Additionally, to check the intensity scaling of HHG cutoff, the pulse intensity enhances from 25 to 100 TW/cm^2, the pulse wavelength is constant and equal to 1500 nm (shown in Fig. S2). In all cases, the pulse duration at full width at half-maximum (FWHM) is equal to 18 fs with a sine-squared envelope shape for the vector potential.

B. Cluster approach ab-initio calculations

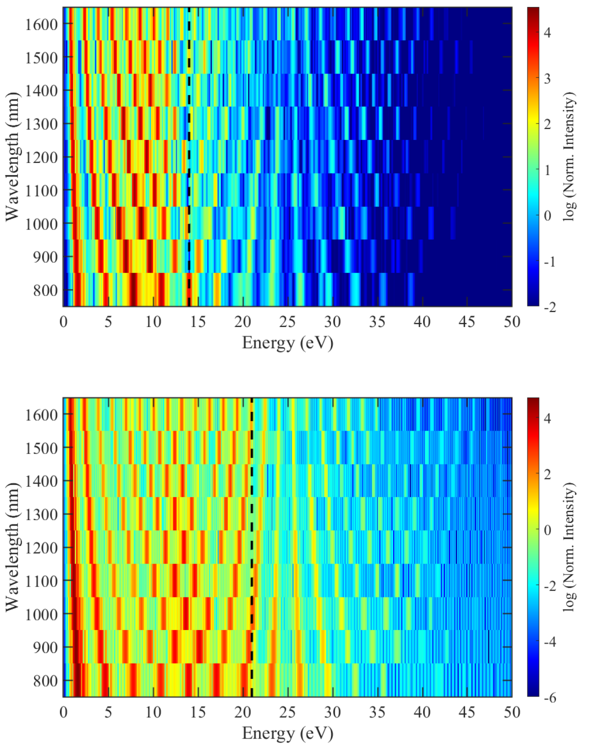

FIG. S3. High-harmonic spectra obtained from calculations on ammonia (NH_3) clusters (top) and methane (CH_4) clusters (bottom) over a broad range of driving wavelengths with a peak intensity of 50 TW/cm^2.

The numerical approach based on clusters that is utilized in the main text is thoroughly described in ref. [9]. We provide here some additional technical details for the calculations presented in the main text. All calculations were performed using the real-space grid-based code, OCTOPUS [5–7]. Cluster geometries for liquid H_2O were taken from ref. [10] using 43-molecule large clusters for the cut-off scaling calculations, and larger 54-molecule clusters for the calculations with varying liquid density that are more sensitive. Cluster geometries for NH_3 and CH_4 were taken from refs. [11] and [12], respectively, with 48-molecule and 40-molecule sizes, respectively. For each cluster, the ground state was obtained by DFT within the PBE [13] approximation for the XC functional with an added van-der-Waals correction term [14], with a real-space cartesian grid of spacing of 0.4 Bohr and spherical grid boundaries with a radius that extended 15 Bohr beyond the farthest atom from the cluster center. In the next step, the HHG response was calculated by propagating the KS states following the prescription in ref. [9] (by suppressing contributions from the surface states, employing orientation averaging, and utilizing the independent particle approximation (the XC potential is frozen to its ground-state form)). The laser pulse was taken to have a trapezoidal envelope in all calculations with a two-cycle long turn-on and turn-off, and a four-cycle-long flat top. The dipole response was filtered with a super-Gaussian prior to obtaining the HHG spectra.

Figure S3 show high-harmonic spectra obtained with this approach for NH_3 clusters (top) and CH_4 clusters (bottom). Both panels show a cut-off energy E_c that is clearly independent of the wavelength. Ammonia was chosen as an example of a different polar molecule that also forms hydrogen bonds (like water and alochols, studied in the main text) and methane was chosen as an example of an apoloar molecule. The results of Fig. S3 thus show that the wavelength independence of E_c is not specific to the liquids studied in the main text, but appears to be a general property of liquid-phase HHG.

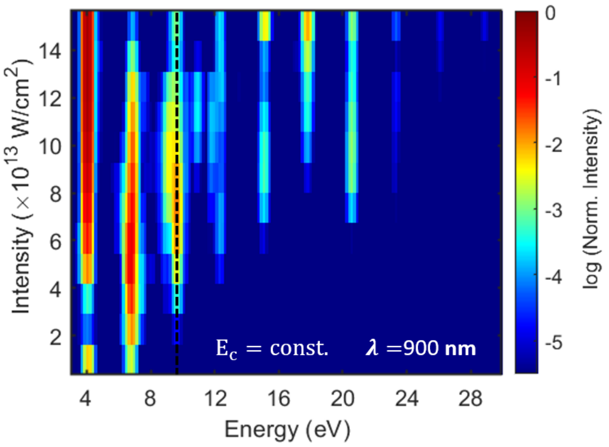

FIG. S4. High-harmonic spectra obtained from calculations on water clusters using a driving wavelength of 900 nm.

To complement the data shown in the main text (Figs. 3C, 3D, 4C), we show here in Fig. S4 the scaling of the high-harmonic spectra with the peak intensity of the driving field with a central wavelength of 900 nm. The figure nicely shows the saturation of the cut-off beyond a threshold intensity.

C. Extended semi-classical calculations

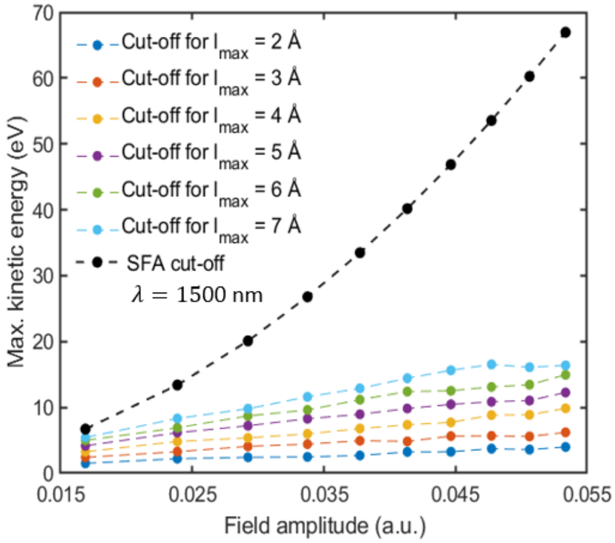

FIG. S5. Scaling of the maximal electron kinetic energy with the peak electric field of the driving pulse.

Based on our suggested extended semi-classical model (main text, Section II.A), we performed semi-analytical calculations for the HHG cutoff vs. the driving laser wavelength and intensity. We also performed calculations with varying l_{max} parameters. In all cases, the Newton equations of motion for the electrons were solved analytically for a given time of ionization, and then represented numerically on a temporal grid to scan for recollisions. The solutions were numerically filtered for each re-colliding trajectory to test whether that particular trajectory extruded beyond l_{max} or not, while the standard TSM was not filtered with this condition. In each case the cutoff was given by the most energetic recolliding trajectory with the added band gap of the liquid. The calculations in Fig. S5 confirm the linear scaling of the maximal kinetic energy with the peak electric-field amplitude of the driving pulse. The onset of saturation is observed at the highest field amplitudes for $l_{\max} = 7$ Å.

D. Semi-classical description of HHG and cutoff scaling including scattering

In this section, we aim at deriving an approximate fully analytical picture that describes the cutoff energy of HHG, as described by our extended semi-analytical semi-classical three-step model. We examine what is the analytical connection between the HHG cutoff and the maximum allowed excursion length of the electron trajectories in the extended model.
Our starting point is the full semi-classical picture discussed in the main text. We consider the classical motion of an electron in an electric field $E(t) = E\cos(\omega t)$, where ω is the frequency of the laser. The magnetic field, as well as the electric field due to the parent ion are neglected and the dipole approximation is employed. The electron is ionized at a time t_i, and we consider the dynamics following this time.

$$x(t) = \frac{qE}{m\omega^2}[\cos(\omega t_i) - \cos(\omega t) - \sin(\omega t_i)\omega(t - t_i)]\,, \quad (1)$$

$$v(t) = \frac{qE}{m\omega}[\sin(\omega t) - \sin(\omega t_i)]\,, \quad (2)$$

where we imposed as initial condition that the trajectories start at $x(t_i) = 0$, with a vanishing velocity ($v(t_i) = 0$).
The time at which the electron returns to its parent ion, referred here as the return time t_r, is obtained by imposing that $x(t_r) = 0$. This gives a first relation, that defines trajectories returning to the parent ion:

$$[\cos(\omega t_i) - \cos(\omega t_r)] = [\sin(\omega t_i)]\omega(t_r - t_i)\,. \quad (3)$$

From the return time, we can obtain the kinetic energy for a trajectory starting at t_i

$$\Delta E_K(t_i) = 2U_p\Big[\sin(\omega t_r) - \sin(\omega t_i)\Big]^2\,. \quad (4)$$

The solution of the Eq. (3) can be performed numerically, see Fig. S6, from which we obtain the well known gas-phase result for the maximum kinetic energy of $\approx 3.17U_p$, corresponding to $\omega t_i \approx 0.305$.

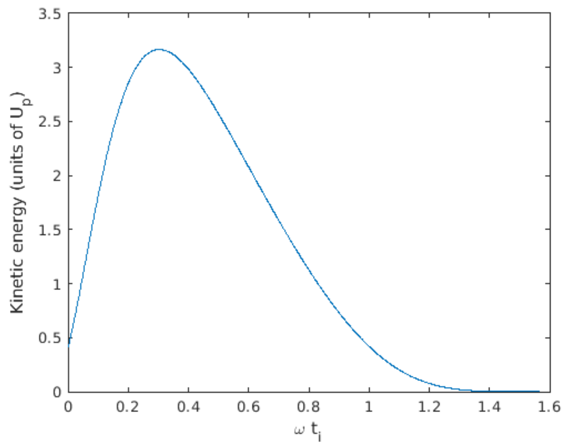

FIG. S6. Evolution of the return kinetic energy versus the ionization phase, obtained from Eq. (4), solving numerically Eq. (3) for each value of t_i.

Let us now consider the case that not all trajectories are allowed, as formulated in our extended model for the liquid. We first need to determine for a given trajectory, determined uniquely by t_i and Eq. (3), what is the maximum excursion.
The maximum excursion is reached when $\partial_t x(t) = v(t) =$

0. This gives us the condition defining the time of maximal excursion t^*:

$$\frac{qE}{m\omega}[\sin(\omega t^*) - \sin(\omega t_i)] = 0 \tag{5}$$

which has a simple analytical solution $\omega t^* = \pi - \omega t_i$. We therefore have an exact expression for the excursion distance l_{exc} for any given ionization time

$$l_{exc}(t_i) = |x(t^*)| = \frac{eE}{m\omega^2}[2\cos(\omega t_i) - \sin(\omega t_i)(\pi - 2\omega t_i)]\,, \tag{6}$$

with $e = |q|$ is the elementary charge. Solving this equation numerically for the most energetic trajectory ($\omega t_i = 0.306$), we find that without limiting the trajectories, the cutoff energy corresponds to an excursion of $\approx 1.145\frac{eE}{m\omega^2}$, which verifies the analytic analysis.

We next consider that the maximal excursion is limited to $l_{\max}$. We can distinguish two cases. As long as $1.145\frac{eE}{m\omega^2}$ is smaller than $l_{\max}$, the kinetic energy gained is given by the usual formula $\Delta E_K \approx 3.17U_p$. Above this threshold, the value of the kinetic energy gain will be modified, as the most energetic trajectory is forbidden, and the cutoff will be reduced. Below, we assume that we are in this regime.
The task is therefore to find the most energetic trajectory that returns to the parent ion, and whose excursion is smaller or equal to $l_{\max}$.
This gives us the condition

$$l_{exc} = \frac{eE}{m\omega^2}[2\cos(\omega t_i) - \sin(\omega t_i)(\pi - 2\omega t_i)] \leq l_{\max}\,. \tag{7}$$

The function $l_{exc}(t_i)$ is a function that decreases monotonously from 2 to 0 over the interval of interest of the ionization time $[0 : \pi/2]$. Therefore a value t_i^* exists such that the condition (7) is satisfied, for all $t_i > t_i^*$ (and smaller than $\pi/2$). Because we are in a regime where not all the trajectories are allowed, this time corresponds to a "short trajectory", meaning that the ionization phase is larger than 0.306, which is the time corresponding to the most energetic trajectory without scattering. Said differently, we only select the short trajectories that have an excursion smaller than $l_{\max}$. Now, we also know that the return kinetic energy decreases monotonously for the short trajectories (see Fig. S6). Therefore, the critical time t_i^* corresponds to the highest kinetic energy in our scattering model.
This ionization time that corresponds to the most energetic trajectory with an excursion smaller or equal to $l_{\max}$ is given by the relation

$$[2\cos(\omega t_i^*) - \sin(\omega t_i^*)(\pi - 2\omega t_i^*)] = L_{\rm red}\,, \tag{8}$$

where we define the dimensionless quantity $L_{\rm red} = \frac{m\omega^2 l_{\max}}{eE}$.
We therefore have two defining equations, Eq. (8) and Eq. (3), where the first one determines the ionization time as a function of the reduced maximum excursion $L_{\rm red}$, and the second one that determines the corresponding return time.

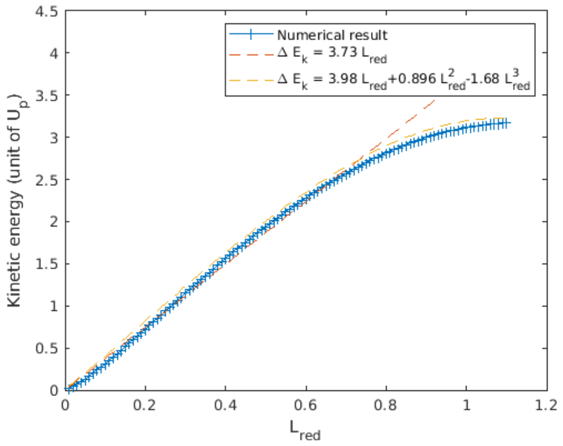

FIG. S7. Evolution of the return kinetic energy versus $L_{\rm red}$. The linear and cubic fits are also shown.

We have solved numerically these two equations: for each value of $L_{\rm red}$, we can find the numerical solution of Eq. (8) and then use this value to numerically solve Eq. (3). The result of the kinetic energy (in units of U_p) as a function of $L_{\rm red}$ is shown in Fig. S7. For not too large values of $L_{\rm red}$, this is well approximated by a linear function. In this regime, which is the one relevant for our experiment, we find that

$$\Delta E_K(\omega t_i) \approx 3.73 U_p L_{\rm red} = \frac{3.73}{4} eEl_{\max} \tag{9}$$

From this result, we clearly see the role of introducing a mean-free path in our model. The cutoff becomes independent of the wavelength and is linear with the electric field. The comparison between the full numerical result and the linear fit is shown for different laser parameters in Fig. S8, showing that for our range of parameters, the approximate linear scaling law matches very well the exact numerical result of our model.

S4. INTENSITY DEPENDENCE OF LIQUID-HHG CUTOFF

In the main text we concluded that the liquid-HHG cutoff was independent of the driving wavelength. However, since the HHG measurements for different wavelengths slightly vary in the driving peak laser intensity (because the threshold intensity of plasma generation depends on the laser wavelength), this conclusion is only valid if the liquid-HHG cutoff also weakly depends on the driver peak intensity in these conditions. We here show that this is indeed the case with both measurements and calculations, which validate that the HHG spectra from liquids is independent of the driving wavelength.

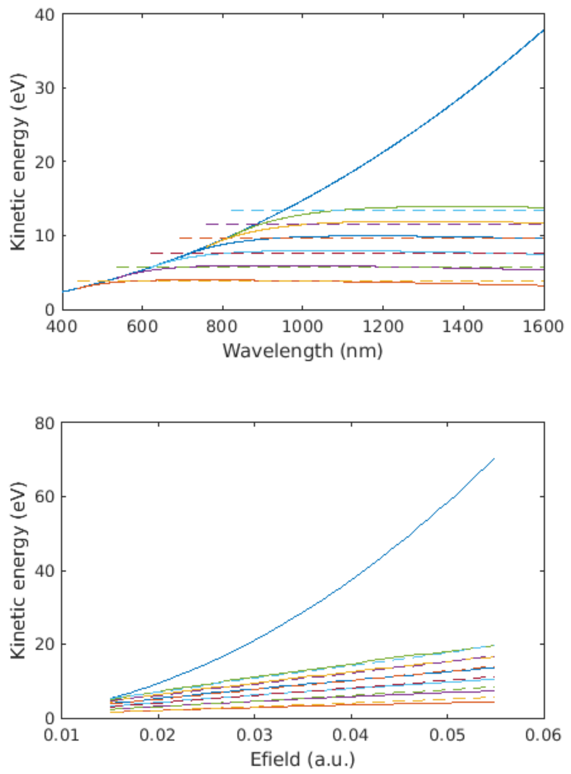

FIG. S8. Evolution of the return kinetic energy versus electric field (left panel) and wavelength(right panel). The parabola correspond to the result without scattering, whereas the solid lines correspond to the result including scattering, for values of $l_{\max} \in \{2, 4, 6, 8, 10, 12, 14\}$ Bohr. The dashed lines correspond to the same results, but obtained using the simpler formula $\Delta E_K = 3.73 U_p L_{\rm red}$.

S5. COMPARATIVE STUDY OF CUT-OFF ENERGY WITH DIFFERENT TRAJECTORY-TRUNCATION METHODS

To study the dependence of the harmonic energy on the implementation of the electron mean free paths, we compare the cut-off energies obtained from different truncation methods. Fig. S9 shows the variation of harmonic yield (i.e. the number of returning trajectories) as a function of the kinetic energy. The analysis is run for different models to find the harmonic yield dependence on the harmonic energy due to the energy dependent $\lambda_{\rm MFP}$. The simulations are performed with a 1500 nm light of 1.6V/Å electric field amplitude. The purple solid line denotes the gas-phase classical SFA model where all trajectories upto the cut-off return with equal probability. The red circles indicate the trajectory model where returning trajectories with excursion lengths greater than 3.1 Å are rejected. The maximum kinetic energy is therefore reduced to ∼2 eV as opposed to 25 eV (SFA). However, in reality trajectories with trajectory lengths extending beyond the $\lambda_{\rm MFP}$ are expected to return but with exponentially decaying probability. Indeed, the probability that a particle is scattered between the travelled distance l and $l + dl$ is given by

$$d\mathcal{P}(l) = \frac{1}{\lambda_{\rm MFP}} e^{-l/\lambda_{\rm MFP}} dl\,, \tag{10}$$

where $\lambda_{\rm MFP}$ is the mean free path of the particle. From this definition, we can compute the probably that a particle is getting scattered after a travelled distance L, given by the travelled distance at the returned time. This is given by

$$\mathcal{P}(L) = \int_0^L \frac{1}{\lambda_{\rm MFP}} e^{-l/\lambda_{\rm MFP}} dl = (1 - e^{-L/\lambda_{\rm MFP}})\,. \tag{11}$$

As L grows, the probability converges to one, as one would naturally expect. In this treatment, we have assumed that the mean free path is independent of the energy of the particle, and therefore the scattering probably only depends on the travelled distance.
It is however possible to go beyond this and to take into account the whole variation of the mean free path along the path of the electron, as we know analytically the trajectories. For this, we convert the integral over the travelled distance into an integral over the time, using the fact that the travelled distance at a point in time t is given by $l(t) = \int_{t_i}^{t} |v(t')|dt'$.

$$\mathcal{P}(L) = \int_{t_i}^{t_r} \frac{1}{\lambda_{\rm MFP}(E_K(t))} e^{-l(t)/\lambda_{\rm MFP}(E_K(t))} |v(t)| dt\,. \tag{12}$$

The yellow line indicates further sophistication of the trajectory model as given by equation (11). This model considers exponential damping by a fixed λ_{MFP} value of 3.1 Å. Finally the blue solid line indicates the returning probability for trajectories calculated considering energy-dependent MFP given by equation (12). We observe that even with the statistical Monte-Carlo-like energy-dependent MFP model (solid blue line), the harmonic yield (returning probability) varies slowly up to $\sim$ 2 eV falling to only 0.5 the value with respect to the hard-cut off and the SFA model. Beyond $\sim$ 2 eV the harmonic yield falls sharply indicating the onset of the cut-off region. This is in considerable agreement with the simplest hard-cut-off model. As a result, the harmonic yield below cut-off does not change considerably to extract the energy-dependent λ_{MFP}(E) with reasonable accuracy taking into account the effect of experimental parameters. Beyond the cut-off energy there is a significant dependence of the harmonic yield on the harmonic energy resulting from λ_{MFP}(E). However, the above cut-off harmonics also have contributions from back scattering and nearest-neighbor recombination as discussed previously. Thus isolating the effect of the energy-dependent λ_{MFP} only for above-cut-off harmonics is beyond the scope of this manuscript.

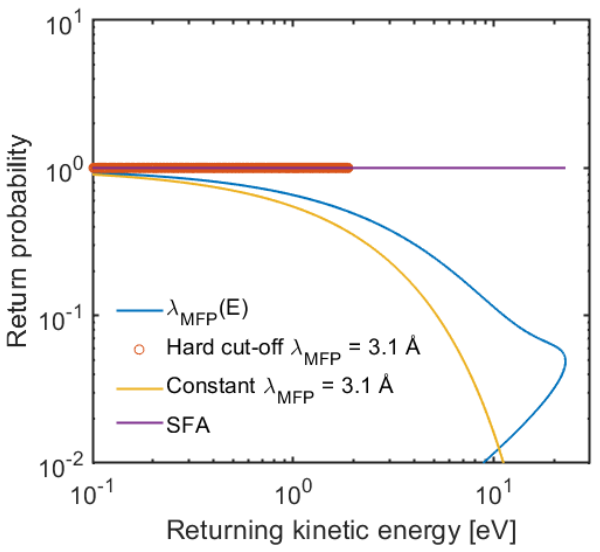

FIG. S9. Shows a comparison of the returning probability of trajectories with different kinetic energies for different variations of the trajectory based model. The purple solid line denotes the gas-phase classical SFA model where all trajectories upto the cut-off return with equal probability. The red circles indicate the trajectory model where returning trajectories with excursion length greater than 3.1 Å are rejected. The maximum kinetic energy is therefore reduced to 2 eV as opposed to 25 eV(SFA). The yellow line indicates further sophistication of the trajectory model where each returning trajectory is modulated by a scattering amplitude of exp(-l_{exc}/3.1Å). This model considers exponential damping by a fixed λ_{MFP} value of 3.1 Å. Finally the blue solid line indicates the returning probability for trajectories calculated considering energy dependent MFP. In this model at each time step of each trajectory, based on their instantaneous kinetic energy, a scattering amplitude of exp(-$l_{exc}/\lambda_{MFP}(E)$) is applied to the returning probability.

S6. ANALYTICAL TREATMENT OF THE PROPAGATION EFFECT

Our goal is to describe how the laser-induced current density lead light emission at a given frequency propagate in a medium, cross the surface of the material and reach the detector. We first calculate the $\mathbf{E}$ and $\mathbf{B}$ fields that result from given source terms. Our approach is quite similar to the approach of Ref. 15, that we generalize here to not only treat the polarization, but also the free charge current and densities, denoted respectively $\rho(\mathbf{r},t)$ and $\mathbf{J}$. In the context of high-harmonic generation in solids, it is mandatory to keep these terms, as the free-charge current ($\mathbf{J}$) describes the intraband contribution while the polarization $\mathbf{P}$, describes the interband contribution.

We begin with the macroscopic Maxwell equations for an homogeneous system

$$\begin{aligned} \boldsymbol{\nabla}\mathbf{D}(\mathbf{r},t) &= 4\pi\rho(\mathbf{r},t) \\ c\boldsymbol{\nabla}\times\mathbf{H}(\mathbf{r},t) - \frac{\partial\mathbf{D}}{\partial t}(\mathbf{r},t) &= 4\pi\mathbf{J}(\mathbf{r},t), \\ \boldsymbol{\nabla}\mathbf{B}(\mathbf{r},t) &= 0, \\ c\boldsymbol{\nabla}\times\mathbf{E}(\mathbf{r},t) + \frac{\partial\mathbf{B}}{\partial t}(\mathbf{r},t) &= 0, \end{aligned} \quad (13)$$

where

$$\begin{aligned} \mathbf{D}(\mathbf{r},t) &= \mathbf{E}(\mathbf{r},t) + 4\pi\mathbf{P}_{\mathrm{tot}}(\mathbf{r},t), \\ \mathbf{H}(\mathbf{r},t) &= \mathbf{B}(\mathbf{r},t) - 4\pi\mathbf{M}_{\mathrm{tot}}(\mathbf{r},t). \end{aligned} \quad (14)$$

The total macroscopic polarization, $\mathbf{P}_{\mathrm{tot}}$, and magnetization, $\mathbf{M}_{\mathrm{tot}}$, have in frequency space the following expression

$$\begin{aligned} \mathbf{P}_{\mathrm{tot}}(\mathbf{r},\omega) &= \chi_e(\omega)\mathbf{E}(\mathbf{r},\omega) + \mathbf{P}_{\mathrm{nl}}(\mathbf{r},\omega) \\ \mathbf{M}_{\mathrm{tot}}(\mathbf{r},\omega) &= \chi_b(\omega)\mathbf{B}(\mathbf{r},\omega) + \mathbf{M}_{\mathrm{nl}}(\mathbf{r},\omega), \end{aligned} \quad (15)$$

where the macroscopic susceptibilities χ_e and χ_b describe the linear response of the medium, and are directly related to the macroscopic dielectric and the permeability tensors of the system

$$\begin{aligned} \epsilon(\omega) &= 1 + 4\pi\chi_e(\omega), \\ \mu^{-1}(\omega) &= 1 - 4\pi\chi_b(\omega). \end{aligned}$$

Note that as we are interested here in systems which are homogeneous at equilibrium, these quantities do not depend on space. The remaining polarization $\mathbf{P}_{\mathrm{nl}}(\mathbf{r},\omega)$ (magnetization $\mathbf{M}_{\mathrm{nl}}(\mathbf{r},\omega)$) term corresponds to the non-linear contributions to the total polarization (magnetization).

Rewriting the Maxwell equations only in terms of $\mathbf{E}$

and $\mathbf{B}$ fields, we obtain

$$\begin{aligned} \epsilon(\omega)\boldsymbol{\nabla}\mathbf{E}(\mathbf{r},\omega) &= 4\pi n_{\mathrm{tot}}(\mathbf{r},\omega) \\ c\boldsymbol{\nabla}\times\mathbf{B}(\mathbf{r},\omega) - i\omega\epsilon(\omega)\mu(\omega)\mathbf{E}(\mathbf{r},\omega) &= 4\pi\mu(\omega)\mathbf{j}_{\mathrm{tot}}(\mathbf{r},\omega), \\ \boldsymbol{\nabla}\mathbf{B}(\mathbf{r},\omega) &= 0, \\ c\boldsymbol{\nabla}\times\mathbf{E}(\mathbf{r},\omega) + i\omega\mathbf{B}(\mathbf{r},\omega) &= 0, \end{aligned} \quad (16)$$

where we defined the total macroscopic nonlinear current and charge densities as

$$\begin{aligned} n_{\mathrm{nl}}(\mathbf{r},\omega) &= \rho(\mathbf{r},\omega) - \boldsymbol{\nabla}\mathbf{P}_{\mathrm{nl}}(\mathbf{r},\omega), \\ \mathbf{j}_{\mathrm{nl}}(\mathbf{r},\omega) &= \mathbf{J}(\mathbf{r},\omega) + i\omega\mathbf{P}_{\mathrm{nl}}(\mathbf{r},\omega) + c\boldsymbol{\nabla}\times\mathbf{M}_{\mathrm{nl}}(\mathbf{r},\omega). \end{aligned} \quad (17)$$

These are the source terms induced by the strong incoming laser, which in term induce the harmonic fields.

There are many situations where the spatial variations of the current cannot be neglected. Due to the multiphoton absorption, the pump laser is depleted, which implies a spatial variation of the laser intensity, and hence of the generated current along the propagation direction. As the laser-matter interaction is highly nonlinear in the case of high-order harmonic generation, many effects have to be taken into account when describing the propagation of the pump laser in the matter, such as self-focusing effects [16], which is caused by the third-order non-linearity of the medium. Moreover, due to surface roughness, nano-structuration of the surface, or possible metalization of the first few atomic layers at the incident surface, the incoming laser can excite surface currents and surface-plasmon-polaritons when the field crosses the interface [17]. It is clear that all these phenomena will induce a spatial variation of the pump laser will propagating in the matter, even at normal incidence, and will thus induce a spatial dependence of the generated nonlinear current. However, while our treatment is general and does no require any approximation of the spatial dependence of the current, we will in the following assume that we have a film which is thin compared to the Rayleigh length of the driving pulse, and neglect the spatial dependence and propagation effects of the incoming laser field.

We now proceed, following the approach of Ref. 15 and we first give the solution of the homogeneous problem defined by Eqs. 16, without considering the presence of the surface. In the view of treating a surface, we construct plane-wave solutions of Eqs. 16 which have a real momentum in the (xy) plane of the surface. [15] The z-axis is chosen to be perpendicular to the surface plane.

There is therefore two possible wave-vectors associated

$$\begin{aligned} \mathbf{q}_+(\omega) &= q_{||}(\omega)\hat{\boldsymbol{\kappa}} + q_\perp(\omega)\hat{\mathbf{z}}\,, \\ \mathbf{q}_-(\omega) &= q_{||}(\omega)\hat{\boldsymbol{\kappa}} - q_\perp(\omega)\hat{\mathbf{z}}\,, \end{aligned}$$

where $\hat{\boldsymbol{\kappa}}$ is the in-plane unit vector of the form $\hat{\boldsymbol{\kappa}} = \cos(\phi)\hat{\mathbf{x}}+\sin(\phi)\hat{\mathbf{y}}$, ϕ being the azimuthal angle, and $q_\perp(\omega)$ is the out-of-plane part of the wave-vector.

Here $q(\omega)^2 = \mu(\omega)\epsilon(\omega)\left(\frac{\omega}{c}\right)^2 = n(\omega)^2\left(\frac{\omega}{c}\right)^2$. and $q_\perp(\omega) = \left(\mu(\omega)\epsilon(\omega)\frac{\omega^2}{c^2} - q_{||}^2(\omega)\right)^{1/2} = \frac{\omega}{c}\left(\mu(\omega)\epsilon(\omega) - \sin^2(\theta)\right)^{1/2}$,

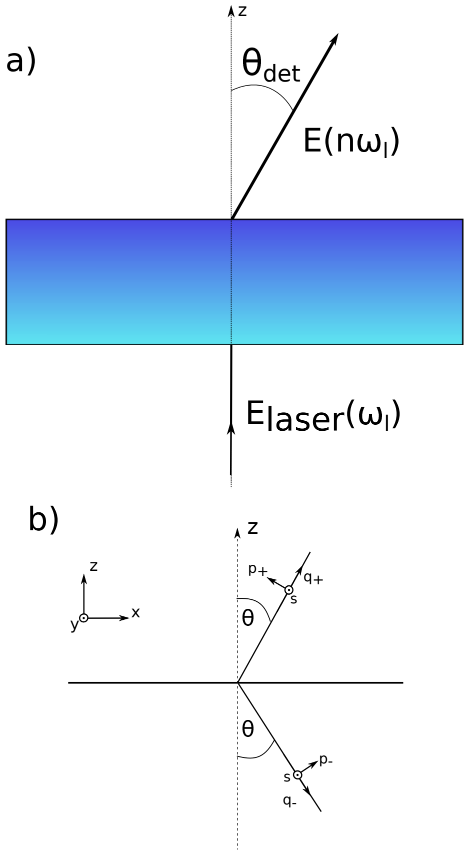

FIG. S10. (a)The experimental geometry considered in this work. The incoming laser field $\mathbf{E}_{\mathrm{laser}}$ is at normal incidence. (b) Coordinate systems used to describe propagation of harmonics. The unit vectors and the link between the different coordinate systems are given in the text. The sketch is presented here for an azimuthal angle $\phi = 0$. Adapted from Ref. 18

$q_\perp$ being chosen to have $\mathrm{Im}[q_\perp] \geq 0$ and $\mathrm{Re}[q_\perp] \geq 0$ if $\mathrm{Im}[q_\perp] = 0$. This convention allows us to be sure that the wave associated with $\mathbf{q}_+$ is upward propagating and that the one associated with $\mathbf{q}_-$ is downward-propagating. [15, 18] We thus refer to the solutions of the homogeneous problem associated with $\mathbf{q}_+$ (respec. $\mathbf{q}_-$) as the upward-propagating (respec. downward-propagating) solutions.

The treatment of the problem represented by equation Eqs. 16 if greatly simplified if we introduce the s and p polarizations, defined by the following unit vectors

$$\begin{aligned} \hat{\mathbf{s}} &= \hat{\boldsymbol{\kappa}} \times \hat{\mathbf{z}}\,, \\ \hat{\mathbf{p}}_\pm(\omega) &= \frac{(q_{||}(\omega)\hat{\mathbf{z}} \mp q_\perp(\omega)\hat{\boldsymbol{\kappa}})}{q(\omega)}. \end{aligned}$$

This is illustrated in Fig. S10(b). For conciseness, we have omitted the frequency dependence here.

One can easily check that we have the following relations

$$\begin{aligned}\hat{\mathbf{s}} \times \hat{\mathbf{q}}_{\pm}(\omega) =& \hat{\mathbf{p}}_{\pm}(\omega)\,,\\ \hat{\mathbf{q}}_{\pm}(\omega) \times \hat{\mathbf{p}}_{\pm}(\omega) =& \hat{\mathbf{s}}\,,\\ \hat{\mathbf{p}}_{\pm}(\omega) \times \hat{\mathbf{s}} =& \hat{\mathbf{q}}_{\pm}(\omega)\,,\end{aligned}$$

where $\hat{\mathbf{q}}_{\pm}(\omega) = \mathbf{q}_{\pm}(\omega)/q(\omega)$. Thus $(\hat{\mathbf{s}}; \hat{\mathbf{q}}_{+}; \hat{\mathbf{p}}_{+})$ and $(\hat{\mathbf{s}}; \hat{\mathbf{q}}_{-}; \hat{\mathbf{p}}_{-})$ are direct basis.
Therefore, the wave associated with $\hat{\mathbf{q}}_{+}$ (respectively $\hat{\mathbf{q}}_{-}$) only propagates along $\hat{\mathbf{s}}$ and $\hat{\mathbf{p}}_{+}$ (respectively $\hat{\mathbf{p}}_{-}$).
Therefore, the upward-propagating solutions of the homogeneous Maxwell equations is of the form

$$\begin{aligned}\mathbf{E}_{+}(\mathbf{r},\omega) =& \left[E_{s^+}(\omega)\hat{\mathbf{s}} + E_{p^+}(\omega)\hat{\mathbf{p}}_{+}(\omega)\right] e^{i\mathbf{q}_{+}(\omega).\mathbf{r}}\,,\\ \mathbf{B}_{+}(\mathbf{r},\omega) =& n(\omega)\left[E_{p^+}(\omega)\hat{\mathbf{s}} + E_{s^+}(\omega)\hat{\mathbf{p}}_{+}(\omega)\right] e^{i\mathbf{q}_{+}(\omega).\mathbf{r}}\,.\end{aligned} \tag{18}$$

The downward-propagating solution as the same form and is not reported here for conciseness.
Knowing the solution of the homogeneous problem, we can now search for the solution of the full problem, described by Eqs. 16. The search of the solution of Eqs. 16 proceed in two steps. We first find the solution associated with source terms of the form [15]

$$\mathbf{j}_{\rm tot}(\mathbf{r},\omega) = \mathbf{j}_{\rm tot}(q_{||}, z_0, \omega)\delta(z - z_0)e^{iq_{||}\cdot\mathbf{r}_{||}}\,, \tag{19}$$

with $\mathbf{r}_{||} = (x, y)$. This corresponds to a sheet of current and density located in the plane $z = z_0$ and having a in-plane wave-vector $q_{||}(\omega)$. The physical solution of the Maxwell equations, for such a source term located at $z = z_0$ (which excludes exponentially diverging waves) still fulfill the homogeneous Maxwell equations for $z \neq z_0$ and therefore reads [15]

$$\begin{aligned}\mathbf{E}(\mathbf{r};\omega) = \Big[&\mathbf{E}_{+}(\mathbf{r};\omega)\Theta(z - z_0)e^{-iq_{\perp}(\omega)z_0}\\ &+\mathbf{E}_{-}(\mathbf{r};\omega)\Theta(z_0 - z)e^{iq_{\perp}(\omega)z_0}\\ &+\mathcal{E}(\omega)\delta(z - z_0)e^{iq_{||}(\omega).\mathbf{r}_{||}}\Big]\,,\end{aligned} \tag{20}$$

and a similar expression for the magnetic field, with here $\Theta(z)$ being the Heaviside function.
The determination of the coefficients $E_{s^\pm}(\omega)$ and $E_{p^\pm}(\omega)$ of Eq. (18) proceed exactly as explained in details in Ref. 15, and is therefore not reported here. After some algebra, we obtain that

$$\begin{aligned}E_{s\pm} =& \frac{2\pi\mu(\omega)\omega}{q^m_{\perp}(\omega)c^2}\hat{\mathbf{s}}.\,\mathbf{j}_{\rm tot}(\mathbf{q}_{||}, z_0, \omega)\,,\\ E_{p\pm} =& \frac{2\pi\mu(\omega)\omega}{q^m_{\perp}(\omega)c^2}\hat{\mathbf{p}}^m_{\pm}(\omega).\,\mathbf{j}_{\rm tot}(\mathbf{q}_{||}, z_0, \omega)\,,\\ \mathcal{B}(\omega) =& 0, \qquad \mathcal{E}(\omega) = -\frac{4\pi i}{\omega\epsilon(\omega)}\hat{\mathbf{z}}\hat{\mathbf{z}}.\,\mathbf{j}_{\rm tot}(\mathbf{q}_{||}, z_0, \omega)\,.\end{aligned} \tag{21}$$

These coefficients determine completely the electric and magnetic fields induced by the source terms of Eqs. (19).

The total electric field induced by one sheet of polarization located at z_0, and evaluated at the position $\mathbf{r}$, with a given in-plane wave-vector $\mathbf{q}_{||}$, denoted $\mathbf{E}_{\mathbf{q}_{||},z_0}$, is thus given by

$$\begin{aligned}\mathbf{E}_{\mathbf{q}_{||},z_0}(\mathbf{r};\omega) = \Big[&\frac{2\pi\mu(\omega)\omega}{q^m_{\perp}(\omega)c^2}\left[\hat{\mathbf{s}}\hat{\mathbf{s}} + \hat{\mathbf{p}}^m_{+}(\omega)\hat{\mathbf{p}}^m_{+}(\omega)\right]\Theta(z - z_0)e^{iq_{\perp}(\omega)(z-z_0)}\\ &+\frac{2\pi\mu(\omega)\omega}{q^m_{\perp}(\omega)c^2}\left[\hat{\mathbf{s}}\hat{\mathbf{s}} + \hat{\mathbf{p}}^m_{-}(\omega)\hat{\mathbf{p}}^m_{-}(\omega)\right]\Theta(z_0 - z)e^{iq_{\perp}(\omega)(z_0-z)} - \frac{4\pi i}{\omega\epsilon(\omega)}\hat{\mathbf{z}}\hat{\mathbf{z}}\delta(z - z_0)\Big]\mathbf{j}_{\rm tot}(\mathbf{q}_{||}, z_0, \omega)e^{iq_{||}\cdot\mathbf{r}_{||}}\,.\end{aligned} \tag{22}$$

A similar expression holds for the $\mathbf{B}$ field. The first term of this expression corresponds to the upward propagating field radiating from the considered source term sheet. The second term is the downward propagating field, and the last one is the local contribution that does not radiate [15].
Exploiting the linearity of Maxwell equations (Eqs. 16), we can obtain the total electric field originating from any general source terms $n_{\rm tot}(\mathbf{r},\omega)$ and $\mathbf{j}_{\rm tot}(\mathbf{r},\omega)$ by integrating over the corresponding in-plane wave-vector and z_0, the latter ranging from z_b to z_t.
The general fields $\mathbf{E}(\mathbf{r};\omega)$ are obtained by summing the individual contributions from all the source terms sheets

$$\mathbf{E}(\mathbf{r};\omega) = \int \frac{d^2\mathbf{q}_{||}}{2\pi}\int_{z_b}^{z_t} dz_0 \mathbf{E}_{\mathbf{q}_{||},z_0}(\mathbf{r},\omega)\,. \tag{23}$$

Similarly, one can also defined the total upward propagating electric field and the magnetic field. After some algebra and using Eq. (19), we obtain

$$\begin{aligned}\mathbf{E}_{+}(\mathbf{r};\omega) =& \frac{2\pi\mu(\omega)\omega}{q^m_{\perp}(\omega)c^2}\left[\hat{\mathbf{s}}\hat{\mathbf{s}} + \hat{\mathbf{p}}^m_{+}(\omega)\hat{\mathbf{p}}^m_{+}(\omega)\right]\\ &\times\int_{z_b}^{z} dz_0 \mathbf{j}_{\rm tot}(\mathbf{r}_{||}, z_0, \omega)e^{iq_{\perp}(\omega)(z-z_0)}.\end{aligned} \tag{24}$$

Similar expressions are obtained for the downward propagating electric field and the magnetic upward/downward propagating fields. In the following, we assume that we can neglect the in-plane position dependence of the electronic current. Thus

$$\begin{aligned}\mathbf{E}_{+}(z;\omega) =& \frac{2\pi\mu(\omega)\omega}{q^m_{\perp}(\omega)c^2}\left[\hat{\mathbf{s}}\hat{\mathbf{s}} + \hat{\mathbf{p}}^m_{+}(\omega)\hat{\mathbf{p}}^m_{+}(\omega)\right]\\ &\times\int_{z_b}^{z} dz_0 \mathbf{j}_{\rm tot}(z_0, \omega)e^{iq_{\perp}(\omega)(z-z_0)}.\end{aligned} \tag{25}$$

A. Measured intensity for thick films

From the previous results, we can now obtain the expression of the radiated harmonic field in the entire system. However, we are only interested in the transmitted harmonic field, which corresponds to the to the electric field in vacuum, measured at some value of z such that $z > z_t$. As we are interested by a macroscopic treatment, the surfaces are modeled by abrupt surfaces with effective Fresnel coefficients. These effective coefficients can be always chosen such that they include the microscopic details of the surfaces. At each interface, a Maxwell saltus occur and only the in-plane component of the electric field's wave-vector is conserved. Therefore, choosing the polarization and wave-vector of the incoming field determines uniquely the fields in vacuum.
We first neglect the multiple reflection, assuming a thick slab. The case of a thin-film with multiple reflections is discussed in the next section.

Knowing the expression of the upward-propagating harmonic fields, we can now look at the expression of the transmitted harmonic light in vacuum.
The harmonic electric field emitted by the crystal is considered to be weak enough to not induce new nonlinear effects, and to have its propagation well described by linear optics laws. At the interface with the vacuum, the field is therefore modified according to the Fresnel coefficients. The harmonic electric field that reaches the detector is thus $\mathbf{E}_{\rm out}(z;n\omega_l) = \mathbf{E}_{\rm out}(z=z_t^+;n\omega_l)e^{iq_\perp^v(n\omega_l)(z-z_t)} = \mathbf{E}_{\rm out}(n\omega_l)e^{iq_\perp^v(n\omega_l)(z-z_t)}$, with

$$\mathbf{E}_{\rm out}(n\omega_l) = \hat{\mathbf{e}}^{n\omega_l}.\mathbf{E}_+(z_t^-;n\omega_l)\,, \tag{26}$$

where $\mathbf{E}_+(z_t^-;n\omega_l)$ is the upward propagating field computing just below the surface. Here, we have defined $\hat{\mathbf{e}}^{n\omega_l} = \hat{\mathbf{e}}^{\mathbf{out}}(n\omega_l)\left[\hat{\mathbf{s}}t^s_{mv}(n\omega_l)\hat{\mathbf{s}} + \hat{\mathbf{p}}^v_+(n\omega_l)t^p_{mv}(n\omega_l)\hat{\mathbf{p}}^m_+(n\omega_l)\right]$, $\hat{\mathbf{e}}^{\mathbf{out}}(n\omega_l)$ being the measured polarization. Note that the vectors $\hat{\mathbf{p}}^i_+(n\omega_l)$ are defined with the detection angle $\theta_{\rm det}$ and not the incidence angle.

If we assume no pump depletion and neglect nonlinear effects in the pump propagation, we can drop position dependence in source term, the electronic current, and replace it by the power n of the phase of the incoming laser, which leads to

$$\begin{aligned}\mathbf{E}_{\rm out}(n\omega_l) &= \frac{2\pi\mu(n\omega_l)n\omega_l}{q_\perp^m(n\omega_l)c^2}\hat{\mathbf{e}}^{n\omega_l}.\mathbf{j}_{\rm tot}(n\omega_l)e^{i(q_\perp(n\omega_l)z_t - nq_\perp(\omega_l)z_b)}\int_{z_b}^{z_t}dz_0 e^{i(nq_\perp(\omega_l)-q_\perp(n\omega_l))z_0}\\ &= \frac{2\pi\mu(n\omega_l)n\omega_l}{q_\perp^m(n\omega_l)c^2}\hat{\mathbf{e}}^{n\omega_l}.\mathbf{j}_{\rm tot}(n\omega_l)i\frac{e^{iq_\perp(n\omega_l)L}-e^{inq_\perp(\omega_l)L}}{(nq_\perp(\omega_l)-q_\perp(n\omega_l))}\,,\end{aligned} \tag{27}$$

where the last term is the phase-matching term and $L = z_t - z_b$. This implies here that we are in the perturbative regime, i.e., that the n-th harmonic scales as the n-th power of the electric field. The modification of the refractive index at the incoming frequency, due to the Kerr effect for instance, can still be taken into account in $q_\perp(\omega_l)$. Note that experimental values points toward a dependence in the electric field of the n-th harmonic much lower that the n-th power of the electric field, and one might replace $nq_\perp(\omega_l)$ by $n_{\rm eff}q_\perp(\omega_l)$ where $n_{\rm eff}$ is an effective number for all harmonic order. From this expression, the measured intensity of the harmonic field is given by the usual relation $I(n\omega_l) = \frac{c\epsilon_0}{2}|\mathbf{E}_{\rm out}(n\omega_l)|^2$.
One special case of interest is normal incidence. As we assumed a cubic, non-magnetic crystal, a $s(p)$-polarized driving field lead to a $s(p)$-polarized current, and thus $I_{pp}(n\omega_l) = I_{ss}(n\omega_l) = I(n\omega_l)$, with

$$I(n\omega_l) = \frac{2\epsilon_0\pi^2}{c\epsilon(n\omega_l)}\left|\hat{\mathbf{e}}^{n\omega_l}.\mathbf{j}_{\rm tot}(n\omega_l)\right|^2\left|\frac{e^{iq_\perp(n\omega_l)L}-e^{inq_\perp(\omega_l)L}}{(nq_\perp(\omega_l)-q_\perp(n\omega_l))}\right|^2 \tag{28}$$

This expression is the generalization of the typical phase-matching expression that one would obtain from perturbative non-linear optic to the n-th order harmonic generation from a slab of matter. It contains the propagation of the incoming light pulse from z_b to z and then the subsequent propagation of the emitted light from z to z_t, for all possible values of z.

B. Measured intensity for thin films

In the case of a thin-film, it is not possible to neglect the multiple reflections of the harmonics inside the material. In this case, Eq. 26 has to be modified, to account for internal reflections. The resulting total outgoing field is composed of the transmitted upward propagating field, plus the multiple reflection of the upward and downward propagating waves.

$$\mathbf{E}_{\rm out}(n\omega_l) = \frac{2\pi\mu(n\omega_l)n\omega_l}{q_\perp^m(n\omega_l)c^2}\hat{\mathbf{e}}^{n\omega_l}.\int_{z_b}^{z_t} dz_0 \mathbf{j}_{\rm tot}(z_0,n\omega_l)e^{iq_\perp(n\omega_l)(z_t-z_0)}$$
$$+\frac{2\pi\mu(n\omega_l)n\omega_l}{q_\perp(n\omega_l)c^2}\hat{\mathbf{e}}^{n\omega_l}.(\hat{\mathbf{r}}_+.\hat{\mathbf{r}}_-).\sum_{n\geq 1}\left(e^{iq_\perp^m(n\omega_l)L}\right)^{2n}(\hat{\mathbf{r}}_+.\hat{\mathbf{r}}_-)^{(n-1)}.\int_{z_b}^{z_t} dz_0 \mathbf{j}_{\rm tot}(z_0,n\omega_l)e^{iq_\perp(n\omega_l)(z_t-z_0)}$$
$$+\frac{2\pi\mu(n\omega_l)n\omega_l}{q_\perp^m(n\omega_l)c^2}\hat{\mathbf{e}}^{n\omega_l}.\hat{\mathbf{r}}_+.\sum_{n\geq 1}\left(e^{iq_\perp(n\omega_l)L}\right)^{(2n-1)}(\hat{\mathbf{r}}_-.\hat{\mathbf{r}}_+)^{(n-1)}.\int_{z_b}^{z_t} dz_0 \mathbf{j}_{\rm tot}(z_0,n\omega_l)e^{iq_\perp(n\omega_l)(z_0-z_b)} \quad (29)$$

where $L = z_t - z_b$ is the thickness of the thin-film and $\hat{\mathbf{r}}_\pm = [\hat{\mathbf{s}}r^s_{mv}(n\omega_l)\hat{\mathbf{s}} + \hat{\mathbf{p}}^m_\pm(n\omega_l)r^p_{mv}(n\omega_l)\hat{\mathbf{p}}^m_\mp(n\omega_l)]$. Following the same approach as in previous section, we now neglect the position dependence of the current and assume a perturbative dependence on the propagation phase of the incoming laser pulse. In the case of normal incidence, assuming again a cubic non-magnetic material, the s and p polarization give the same result and the intensity of the radiated field is then given by

$$I(n\omega_l) = \frac{2\epsilon_0\pi^2}{c\epsilon(n\omega_l)}|\mathbf{j}_{\rm tot}(n\omega_l)|^2\left|\frac{t_{mv}}{1-e^{2iq_\perp(n\omega_l)L}(r_{mv})^2}\right|^2\left|\frac{e^{iq_\perp(n\omega_l)L}-e^{inq_\perp(\omega_l)L}}{(nq_\perp(\omega_l)-q_\perp(n\omega_l))}+r_{mv}\frac{e^{iq_\perp(n\omega_l)L}-e^{i(nq_\perp(\omega_l)+2q_\perp(\omega_l))L}}{(nq_\perp(\omega_l)+q_\perp(n\omega_l))}\right|^2 \quad (30)$$

Equation 30 formula is the equivalent of Eq. 28 for the case of multiple reflections. We found numerically that in our experimental conditions, the multiple reflections do not play any important role.

C. Results for the propagation effects

We now employed the above model to simulate the effect of light propagation in water jet, and in particular to simulate the effect of re-absorption of the harmonic light by the jet itself. We considered a jet thickness of 1 *mum*. We assumed that the laser generate an harmonic spectrum as in the gas phase at each possible position along the slab, and we employed the experimental dielectric function of water to model light-propagation effects [19]. Due to the reabsorption, we found that the total measured signal, that reaches the detector, builds up within the 200nm close to the exist surface. However, our results, shown in Fig. S11 shows that the propagation effects, including phase-matching effects and light re-absorption, cannot explain the change in the energy cutoff. We also found (not shown) that the multiple reflections of either the incoming light or of the emitted harmonic light are not changing significantly the effect of the propagaition.

S7. EXPERIMENTAL RESULTS FOR DENSITY AND PROPAGATION EFFECTS

To isolate the effects of propagation and density liquid generated high harmonic spectra we experimentally measured jet thickness dependence of the harmonic cut-off and harmonic yield. If the cut-off energy is dependent on the propagation length through the liquid, a change in the jet thickness would show a change in the cut-off energy. The thickness for a flat-jet varies in the vertical direction. However, we have seen for Ethanol liquid (Figure 4(A) of manuscript) that the temperature also changes along the vertical direction. To isolate the effect of only the thickness variation we specifically choose H_2O and the vertical positions of (0.66-1.26 mm from top of jet). The thickness has been measured using a few-cycle 800 nm XUV-XUV high harmonic interference and verified through white light interferometry[1].Figure S13 (A) shows the fractional density change in percentage as a function of the jet position and the corresponding jet thickness. The density change has been calculated from the temperature variation as a function of the jet position, obtained from the calculations of [20] for H_2O flat-jet formed using 40 um nozzle diameter(used for the thickness measurements here)[20]. The corresponding water density has been interpolated from Table 17 of [21]. It is observed that in this vertical scan range, while the thickness changes from 1000 nm to 500 nm the fractional change in density with respect to the 1000 nm data point is only about 0.04%, thus for H_2O and the given jet positions we can clearly say, that whatever cut-off energy shift,in any, we observe should solely be because of the jet thickness variation. Figure S13 (B) shows the harmonic spectrum of H_2O measured for different jet thicknesses using 800 nm driving wavelength. Each harmonic spectra has been normalized to the 5th harmonic (energy 7.76 eV) of 800 nm. The Figure S13 (B) clearly indicates that the harmonic spectra are almost identical, irrespective of the jet thickness. To observe this carefully we plot Figure S13 (C) that shows the ratio of each harmonic signal (Normalized yield summed over an energy bin of 0.5 eV centered around each harmonic peak). The top x axis of Figure S13 (C) indicates the fraction change in density in percentage with respect to the data point at 1000 nm thickness (vertical position 0.66 mm). It is observed that the harmonic ratio varies negligibly as a function of the jet thickness. This observation is in contrast to what we see for ethanol (Figure 4(A) and (Figure S14)), where the fractional density is significantly large and we see a clear variation of the harmonic ratio as a function of the fractional density change. This further supports the mi-

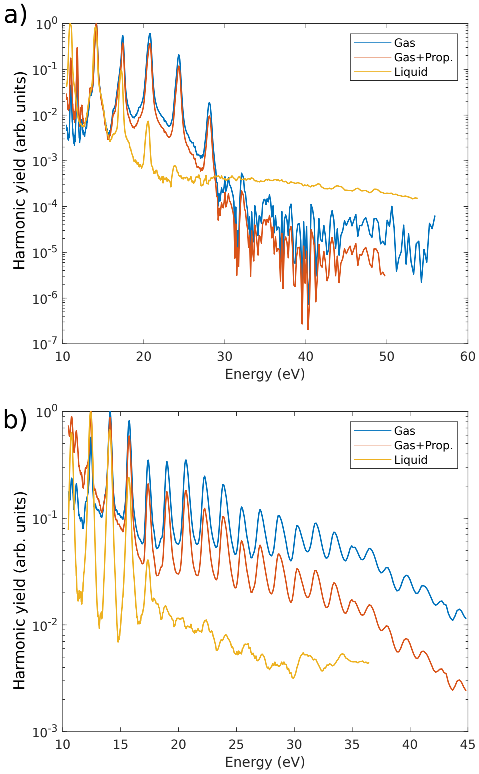

FIG. S11. Effect of propagation for a $1\mu m$ thick slab of liquid water. The emission is assumed to be given by the gas phase for the same laser intensity as for the liquid phase measurement. The effect of harmonic light propagations is computed here for (a) 800 nm driving field, and (b) 1500 nm driving field. In both cases, the change in the energy cutoff cannot be explained by the laser reabsorption.

croscopic origin of the cut-off energy shift, that is now clearly isolated to be a density effect as opposed to an absorption effect of the liquid medium.

We compare these results to the harmonic yields observed for different spectra presented in Fig. 4(A) of the manuscript, where we have normalized each spectrum to the 11 th harmonic of 1800 nm. Figure S14 (A) plots the normalized harmonic yields as a function of harmonic energy for the four different spectra of Fig. 4(A) where the first four energies correspond to the harmonics below cut-off (H11 H13 H15 H17) and the two highest energies (H19 and H21) correspond to harmonics above cut-off. The harmonic yield has been computed by a summation of the harmonic signal within an energy bin of 0.5 eV centered on each harmonic peak. The measured signal is then normalized to the signal of harmonic 11. Figure S14 (B) shows the harmonic ratios obtained for different density variations from the cluster calculations done for a laser wavelength of 900 nm. Comparing Figure S14 (B) with the Figure S14 (A) we see that the harmonics below the cut-off behave similar to what we expect from trajectory simulation, where reducing the fixed λ_{MFP} values (equivalent to increasing density) systematically

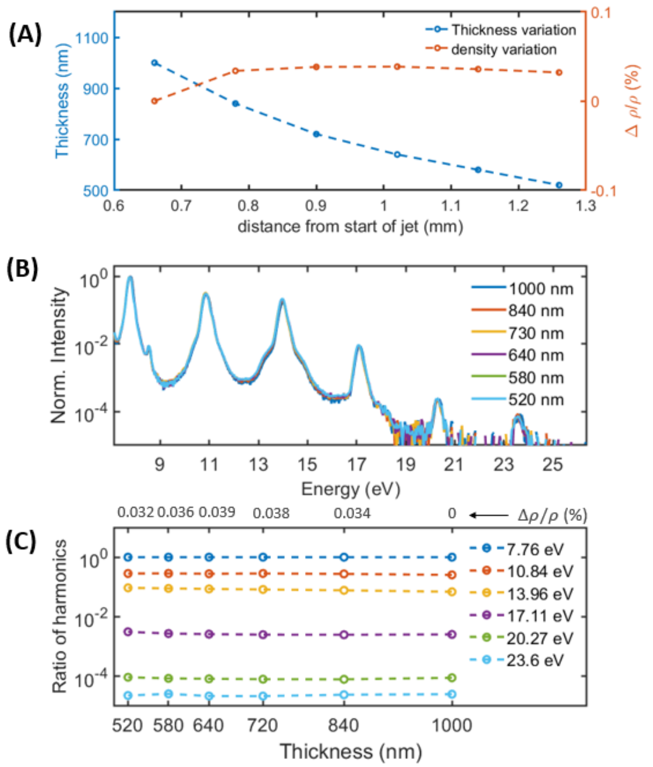

FIG. S12. Effect of jet thickness on the cut-off energy of liquid harmonics of H_2O measured at 800 nm wavelength. **(A)** Shows the fractional density change in percentage as a function of the jet position and the corresponding jet thickness obtained from [1],[20] and [21].**(B)** The harmonic spectrum of H_2O measured for different jet thicknesses using 800 nm driving wavelength. Each harmonic spectra has been normalized to the 5th harmonic (energy 7.76 eV) of 800 nm. **(C)** Ratio of each harmonic signal (Normalized yield summed over an energy bin of 0.5 eV centered around each harmonic peak). The top x axis of Figure S13 **(C)** indicates the fraction change in density in percentage with respect to the data point at 1000 nm thickness (vertical position 0.66 mm).

suppresses the harmonic yields, which essentially emphasizes the validity of the fixed MFP assumption. However for harmonics beyond cut-off (H19 and H21) the trend seems to change increasing in yield for higher densities. This might involve contribution from the energy dependent MFP but harmonics beyond cut-off also have contributions from backscattering and nearest neighbor recombination as discussed previously. Thus isolating the contribution of just one effect is beyond the scope of this manuscript.

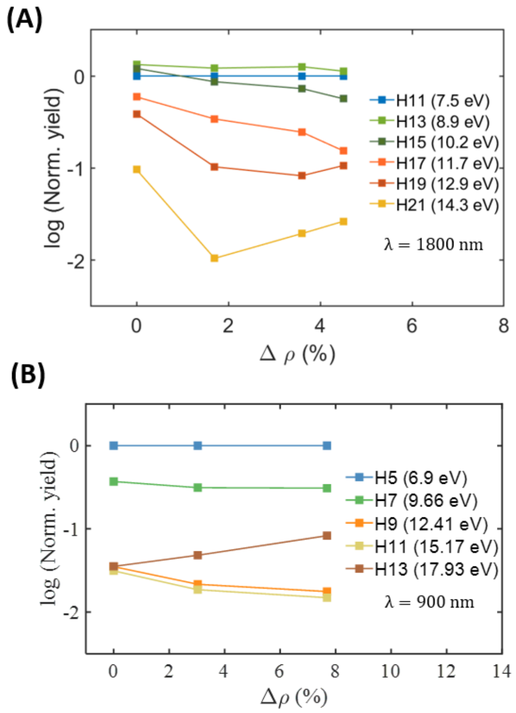

FIG. S13. **(A)** Normalized harmonic yields as a function of harmonic energy for the four different spectra of Fig. 4**(A)** where the first three energy points correspond to the harmonics below cut-off (H11 H13 H15 and H17). **(B)** The harmonic ratios obtained for different density variations from the cluster calculations done for a laser wavelength of 900 nm and intensity of 4× 10^{13} W/cm^2.

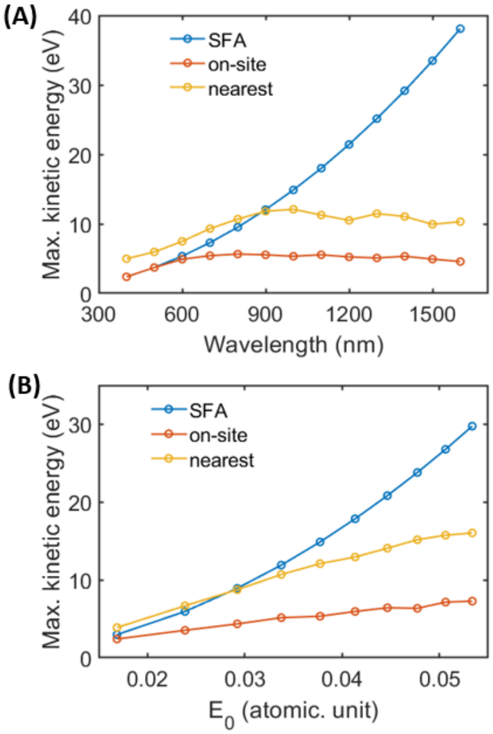

FIG. S14. Comparison of maximum kinetic energies for onsite recombination and nearest-neighbor recombination: **(A)** As a function of wavelengths, calculated at an intensity of $5{\times}10^{13}$ W/cm^2. **(B)** As a function of driving field for 1000 nm driving wavelength.

S8. COMPARISON OF CUT-OFF ENERGY OBTAINED FROM THE SEMI-CLASSICAL SCATTERING MODEL FROM NEAREST NEIGHBOR INTERACTION AND ONSITE INTERACTION

From the perspective of a semi-classical trajectory based model, the energy cutoff obtained from the recombination to the neighboring site is significantly larger than for a wave packet recombining to its parent ion, because of a higher kinetic energy at the collision time. Thus, if recombination channels on neighboring molecules were playing a significant role the cutoff energy would have been almost double than that of the onsite recombination, which contradicts our observations. The figure below shows the comparison of the maximum kinetic energy as a function of laser wavelength (Figure S15 A) and as a function of the laser field (Figure S15 B) for the on-site recombination and nearest neighbor recombination as calculated from the semi-classical scattering model. In addition to the nearest neighbor interaction having higher cut-off than the onsite interaction, we also observe that for wavelengths below 900 nm the nearest neighbor interaction should ideally give a cut-off higher than the SFA (for the gas generated harmonics). But from the data presented in Figure S1 of the supplement, this is definitely not the case. Moreover we see that for the nearest neighbor interaction the maximum kinetic energy has a stronger dependence on the electric field than the onsite interaction, which is in contrast to our observations in Figure S1(D).

S9. CHANGE OF THE CUTOFF ENERGY FOR ELLIPTICALLY POLARIZED LIGHT

In the case of elliptically-polarized light of a given polarization ϵ, the semi-classical equation of motion of the electron, ionized at a time t_i, is given by

$$x(t) = \frac{qE}{m\omega^2}[\cos(\omega t_i) - \cos(\omega t) - \sin(\omega t_i)\omega(t - t_i)]\,,$$
$$y(t) = \frac{qE\epsilon}{m\omega^2}[\sin(\omega t_i) - \sin(\omega t)] + v_{0y}(t - t_i)\,,$$
$$v_x(t) = \frac{qE}{m\omega}[\sin(\omega t) - \sin(\omega t_i)]\,,$$
$$v_y(t) = -\frac{qE\epsilon}{m\omega}[\cos(\omega t)] + v_{0y}\,,$$

where we imposed as initial condition that the trajectories start at $x(t_i) = y(t_i) = 0$, with a non-vanishing velocity along the minor-axis direction (y), that guaranties

that the electron returns to its parent ion ($y(t_r) = 0$) at the return time. As in linearly-polarized case, the return time is given by the condition $x(t_r) = 0$. This leads to $v_{0y} = \frac{qE\epsilon}{m\omega^2} \frac{\sin(\omega t_r) - \sin(\omega t_i)}{\omega(t_r - t_i)}$.
From these equation of motions, we considered only returning trajectories which travel a distance $d = \int_{t_i}^{t_r} |\mathbf{v}(t)| dt$ smaller than an effective mean-free-path distance, and computed the corresponding gain in kinetic energy at the return time. This directly allows us to compute the change in cutoff energy for our model with respect to the driver ellipticity, as shown in the main text Figure ED1. We found that the energy cutoff decreases with increasing ellipticity. We note that if we instead would start from a zero velocity, we also obtain a decrease of he energy cutoff with the increasing ellipticity for small ellipticities, and an increase for large ellipticities, because of the unphysical starting point. If we consider instead of the recombination to the parent ion the recollision with the nearest neighboring molecule, we obtain that the increasing ellipticity leads to an increase of the energy cutoff, which is the opposite trend as observed experimentally.

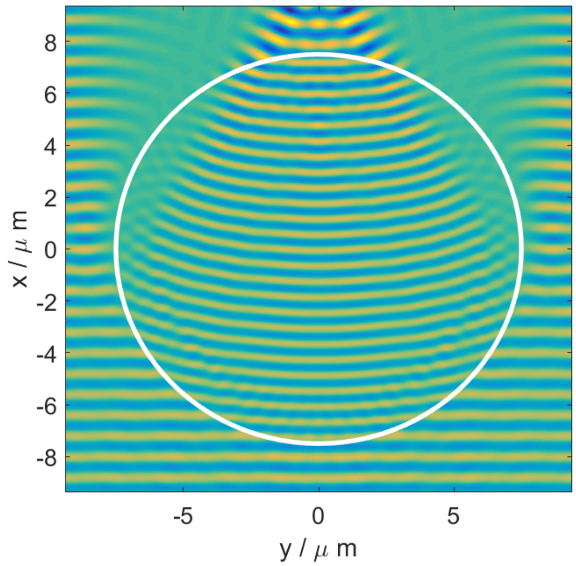

FIG. S15. Numerical simulations of the near-field distribution of an 800 nm wave propagating from bottom to top through a droplet of liquid water with a diameter of 15 μm. The outer boundaries of the liquid-water droplet are indicated with a white line.

S10. COMPARISON WITH A PRIOR WORK ON HHG FROM LIQUID DROPLETS

In this section, we briefly compare and contrast our work with that of Kurz *et al.* [22]. Briefly, our work investigates HHG in liquids at intensities below the optical breakdown limit, whereas Kurz et al. used laser intensities far above this limit. Kurz et al. concluded from their work that HHG was not observed at the density of liquid water. Our work shows the opposite and introduces a spectroscopic technique for determining electron mean-free paths from high-harmonic spectra.
The key differences between the two works are the following:

- In our work, we investigate liquids, and in particular liquid water, at their natural density (i.e. for water, 3.35×10^{22} cm^{-3}). In contrast, Kurz *et al.* did not observe any harmonic radiation at this density, but only at densities below 10^{22} cm^{-3}.

- The systems studied by Kurz *et al.* were exploding plasma droplets, not liquid-water droplets. Indeed, the pump pulses initiating the droplet expansion were strong enough (4.5×10^{14} W.cm^{-2}) to fully ionize the droplets and multiply ionize a dominant fraction of the molecules. Our experimental setup allows us to observe HHG in liquids at their natural density, without the need of a prior expansion caused by an optical breakdown.

- The probe pulses of Kurz *et al.* were so strong (1.4×10^{15} W.cm^{-2}) that they quadruply ionized the water molecules, see for instance Ref. [23]. This implies that Kurz *et al.* did not observe HHG from a dense gas of neutral molecules, but from a highly charged plasma. In sharp contrast, the laser pulses used to drive HHG in our work are 10 to 30 times weaker ($1.5 - 4.5 \times 10^{13}$ W.cm^{-2}), enabling HHG well below the optical-breakdown limit.

- The model employed in our work only superficially resembles that of Kurz *et al.* because its working hypothesis is fundamentally different. Unlike Kurz *et al.*, we do not assume that electron scattering is described by gas-phase scattering cross sections. We instead treat the electron mean-free path as an unknown and show that it can be extracted from the measurements. This working hypothesis is confirmed experimentally in the present work, without any model assumptions, through a measurement of the cut-off as a function of the liquid density (Fig. 4).

Importantly, the conclusion from Kurz *et al.* is that HHG (above the 7th order) in liquid water is impossible. They indeed wrote that "*The HHG process breaks down for densities $\rho > \rho_s$ [red area in Figs. 3(b)–3(d)], which is the density limit derived in Fig. 1. The disappearance of the harmonic radiation is confirmed by the comparison with the spectra in Fig. 3(a), where the harmonic peak structure is lost for densities $\rho > \rho_s$* ". The consequence of this conclusion is that the research field of high-harmonic spectroscopy of liquids and solutions would be an impasse. Our work demonstrates the opposite.

Let us finally propose an explanation of the discrep-

ancy between the conclusion of the work of Kurz *et al.* and our results. This can be explained in terms of the geometry of the targets. Kurz *et al.* used water droplets with an initial diameter of 15 μm. Figure S15 shows numerical simulations of the near-field distribution of an 800-nm wave incident on a sphere of 15 μm diameter. These simulations show that the liquid-water droplet acts as a microlens on the incoming beam, which results in microfocussing of the 800-nm beam right behind the droplet. The consequence for HHG is that the high harmonics will be produced inside the droplet, with wavefronts that follow the curved wavefronts of the 800-nm driving beam. As a consequence, the high harmonics will also microfocus behind the droplet and then diverge. This divergence is so strong that no high harmonic intensity can be expected using a typical flat-field spectrometer as used by Kurz *et al.* (and in our experiments), the detector plane of which is on the order of 1 m away from the HHG source. We believe that this explains why Kurz *et al.* did not observe HHG at the density of liquid water, but our experiments did, because we used (1 μm) thin flat jet with nearly parallel interfaces.

---

[1] Yin, Z., Luu, T. T. & Wörner, H. J. Few-cycle high-harmonic generation in liquids: in-operando thickness measurement of flat microjets. *Journal of Physics: Photonics* **2** (4), 044007 (2020) .

[2] Nourbakhsh, Z., Neufeld, O., Tancogne-Dejean, N. & Rubio, A. An ab initio supercell approach for high-harmonic generation in liquids (2022). arXiv:2212.04177 [cond-mat.mtrl-sci].

[3] Giannozzi, P. *et al.* Advanced capabilities for materials modelling with quantum ESPRESSO. *Journal of Physics: Condensed Matter* **29** (46), 465901 (2017). URL `https://doi.org/10.1088/1361-648x/aa8f79`. doi:10.1088/1361-648x/aa8f79 .

[4] Zhang, Y. & Yang, W. Comment on "generalized gradient approximation made simple". *Phys. Rev. Lett.* **80**, 890–890 (1998). URL `https://link.aps.org/doi/10.1103/PhysRevLett.80.890`. doi:10.1103/PhysRevLett.80.890 .

[5] Marques, M. A., Castro, A., Bertsch, G. F. & Rubio, A. Octopus: a first-principles tool for excited electron–ion dynamics. *Computer Physics Communications* **151** (1), 60–78 (2003) .

[6] Castro, A. *et al.* octopus: a tool for the application of time-dependent density functional theory. *physica status solidi (b)* **243** (11), 2465–2488 (2006) .

[7] Andrade, X. *et al.* Real-space grids and the octopus code as tools for the development of new simulation approaches for electronic systems. *Phys. Chem. Chem. Phys.* **17**, 31371–31396 (2015). doi:10.1039/C5CP00351B .

[8] Andrade, X. *et al.* Modified ehrenfest formalism for efficient large-scale ab initio molecular dynamics. *Journal of Chemical Theory and Computation* **5** (4), 728–742 (2009). URL `https://doi.org/10.1021/ct800518j`. doi:10.1021/ct800518j, pMID: 26609578, https://doi.org/10.1021/ct800518j .

[9] Neufeld, O., Nourbakhsh, Z., Tancogne-Dejean, N. & Rubio, A. Ab initio cluster approach for high harmonic generation in liquids. *Journal of Chemical Theory and Computation* **18** (7), 4117–4126 (2022). doi:10.1021/acs.jctc.2c00235 .

[10] Kazachenko, S. & Thakkar, A. J. Water nanodroplets: Predictions of five model potentials. *The Journal of Chemical Physics* **138** (19), 194302 (2013). URL `https://doi.org/10.1063/1.4804399`. doi:10.1063/1.4804399, https://doi.org/10.1063/1.4804399 .

[11] Orabi, E. A. & Lamoureux, G. Polarizable interaction model for liquid, supercritical, and aqueous ammonia. *Journal of Chemical Theory and Computation* **9** (4), 2035–2051 (2013). URL `https://doi.org/10.1021/ct301123j`. doi:10.1021/ct301123j .

[12] Takeuchi, H. The structural investigation on small methane clusters described by two different potentials. *Computational and Theoretical Chemistry* **986**, 48–56 (2012). doi:https://doi.org/10.1016/j.comptc.2012.02.010 .

[13] Perdew, J. P., Burke, K. & Ernzerhof, M. Generalized gradient approximation made simple. *Physical review letters* **77** (18), 3865 (1996) .

[14] Grimme, S., Antony, J., Ehrlich, S. & Krieg, H. A consistent and accurate ab initio parametrization of density functional dispersion correction (dft-d) for the 94 elements h-pu. *The Journal of Chemical Physics* **132** (15), 154104 (2010). URL `https://doi.org/10.1063/1.3382344`. doi:10.1063/1.3382344 .

[15] Sipe, J. E. New green-function formalism for surface optics. *J. Opt. Soc. Am. B* **4** (4), 481–489 (1987). URL `http://josab.osa.org/abstract.cfm?URI=josab-4-4-481`. doi:10.1364/JOSAB.4.000481 .

[16] Boyd, R. W. in *Nonlinear optics* 161–183 (Taylor & Francis, 2003).

[17] Derrien, T. J.-Y., Krüger, J. & Bonse, J. Properties of surface plasmon polaritons on lossy materials: lifetimes, periods and excitation conditions. *Journal of Optics* **18** (11), 115007. URL `http://stacks.iop.org/2040-8986/18/i=11/a=115007` .

[18] Mizrahi, V. & Sipe, J. E. Phenomenological treatment of surface second-harmonic generation. *J. Opt. Soc. Am. B* **5** (3), 660–667 (1988). URL `http://josab.osa.org/abstract.cfm?URI=josab-5-3-660`. doi:10.1364/JOSAB.5.000660 .

[19] Hayashi, H. & Hiraoka, N. Accurate measurements of dielectric and optical functions of liquid water and liquid benzene in the vuv region (1–100 ev) using small-angle inelastic x-ray scattering. *The Journal of Physical Chemistry B* **119** (17), 5609–5623 (2015) .

[20] Chang, Y.-P., Yin, Z., Balciunas, T., Wörner, H. J. & Wolf, J.-P. Temperature measurements of liquid flat jets in vacuum. *Structural Dynamics* **9** (1), 014901 (2022) .

[21] Patterson, J. B. & Morris, E. C. Measurement of absolute water density, 1 °c to 40 °c. *Metrologia* **31** (4), 277–288 (1994). URL `https://doi.org/10.1088/0026-1394/31/4/001`. doi:10.1088/0026-1394/31/4/001 .

[22] Kurz, H. G. *et al.* Revealing the microscopic real-space

excursion of a laser-driven electron. *Physical Review X* **6** (3), 031029 (2016) .

[23] Liu, H. *et al.* Charge resonance enhanced multiple ionization of h2o molecules in intense laser fields. *Chinese Physics Letters* **32** (6), 063301 (2015) .
